# Organelle-specific crosstalk between calcium and redox signaling in pancreatic β-cells in physiology and type 2 diabetes

**DOI:** 10.3389/fnetp.2026.1864821

**Published:** 2026-08-24

**Authors:** Lucas Rafael Pereira Camara, Eloisa Aparecida Vilas-Boas

**Affiliations:** Department of Clinical and Toxicological Analysis, School of Pharmaceutical Sciences, University of Sao Paulo, Sao Paulo, Brazil

**Keywords:** calcium signaling, insulin secretion, oxidative stress, pancreatic β-cells, redox signaling, type 2 diabetes mellitus

## Abstract

Two of the main intracellular signaling molecules in pancreatic β-cells are calcium (Ca^2+^) and reactive oxygen species (ROS). Ca^2+^ signaling is essential for regulating energy metabolism, insulin secretion, and the modulation of key factors involved in β-cell survival and function. In parallel, redox signaling also affects energy metabolism and enhances glucose-stimulated insulin secretion. Imbalances in Ca^2+^ and redox signaling may contribute to the development of various pathological conditions, including type 2 diabetes mellitus (T2D). T2D is one of the most prevalent diseases worldwide and is characterized by a progressive decline in insulin secretion, typically in the context of obesity and insulin resistance in target tissues. Insulin resistance and poor glycemic control lead to the development of several complications, such as retinopathy, kidney dysfunction, and neuropathy, which negatively impact both quality and life expectancy. Understanding how Ca^2+^ and ROS are modulated during the progression of T2D may aid in the development of pharmacological strategies aimed at preventing β-cell dysfunction or promoting their functional recovery. The two main intracellular Ca^2+^ stores in β-cells are the endoplasmic reticulum (ER) and mitochondria, while two important sources of ROS are mitochondria and NADPH oxidase (NOX) enzymes. It is also known that ROS and Ca^2+^ interact bidirectionally: Ca^2+^ can stimulate oxidative phosphorylation, increasing mitochondrial ROS production; some mitochondrial Ca^2+^ channels are redox-sensitive; certain Ca^2+^-dependent enzymes respond to redox signals; and some NOX isoforms are activated by Ca^2+^. Thus, ROS can modulate Ca^2+^ signaling, while Ca^2+^ signaling is essential for ROS production, establishing a feedback loop that affects β-cell function and survival. The aim of this review is to summarize the main findings on Ca^2+^ and ROS in pancreatic β-cells, addressing each of these signaling systems separately in physiological conditions and in T2D, and subsequently discussing their functional interaction and how these mechanisms function within the islet network. We also highlight the integration of the different intracellular compartments involved in these processes, namely, the ER, mitochondria, and NOX enzymes.

## 1 Introduction

Pancreatic β-cells play a central role in maintaining glucose homeostasis by coupling metabolic signals to insulin secretion. Glucose metabolism triggers electrical activity and Ca^2+^ influx, ultimately driving insulin exocytosis. Ca^2+^ can also be mobilized by intracellular stores, highlighting the contribution of multiple organelles. In parallel, reactive oxygen species (ROS), particularly hydrogen peroxide (H_2_O_2_), have emerged as important modulators of ion channel activity, metabolic pathways, and stimulus-secretion coupling. While physiological levels of ROS contribute to normal β-cell function, excessive ROS production together with disturbances of Ca^2+^ signaling are associated with the development of β-cell dysfunction in pathological conditions such as type 2 diabetes (T2D).

Importantly, Ca^2+^ and redox signaling are not independent processes, but are tightly interconnected across several intracellular compartments. Despite extensive investigation of Ca^2+^ dynamics and ROS signaling individually, their integrated regulation, in an organelle-specific context, remains less clearly defined. A comprehensive understanding of how these systems interact is of great interest to elucidate the mechanisms responsible for β-cell dysfunction in T2D. Notably, most insights into β-cell Ca^2+^ and redox signaling come from rodent islets and cell lines, which do not fully recapitulate human β-cell physiology. We therefore specify the experimental model used and highlight relevant species differences throughout.

Also, β-cell function cannot be fully understood in isolation. Within pancreatic islets, β-cells operate in a highly coordinated multicellular network, where gap junctions and paracrine interactions shape the spatiotemporal dynamics of Ca^2+^ and redox signaling. These signals may extend beyond individual cells, influencing network synchronization and intercellular communication. Disruption of these integrated processes represents a key feature of islet dysfunction in T2D.

In this review, we provide an integrated perspective on Ca^2+^ and ROS signaling in pancreatic β-cells. We first discuss each of these signaling systems separately in physiological conditions and in T2D, and subsequently examine their functional interplay, with particular emphasis on organelle-specific mechanisms. Finally, we address how these processes operate within the islet network and contribute to β-cell dysfunction in T2D.

## 2 Calcium (Ca^2+^) and insulin secretion

Calcium (Ca^2+^) is an ion involved in virtually all fundamental processes in biological systems. It exerts its functions both in the extracellular and intracellular environments. The extracellular concentration of ionized Ca^2+^ (∼1.1–1.3 mM) is approximately 10,000 times higher than the cytosolic concentration (∼100 nM), and the maintenance of this gradient is essential for cellular physiology (Clapham, 2007).

Insulin secretion by pancreatic β-cells is a complex process that occurs in response to an increase in intracellular Ca^2+^ ([Ca^2+^]_i_). This process is highly regulated, and the primary trigger for secretion is the elevation of blood glucose levels, a mechanism known as glucose-stimulated insulin secretion (GSIS) (Henquin, 2000). In both humans and rodents, GSIS exhibits a biphasic pattern: an initial rapid and transient phase (first phase), followed by a slower and sustained increase in insulin secretion (second phase) (Henquin, 2011).

In rodents, the main glucose transporter is GLUT2, characterized by high capacity and low affinity, enabling glucose influx to vary proportionally with circulating concentrations. In contrast, human β-cells predominantly express the GLUT1 isoform, which has a higher affinity for glucose (McCulloch et al., 2011; Mueckler and Thorens, 2013). In general, after entering the cell, glucose is metabolized through glycolysis, culminating in the formation of pyruvate, which is converted into acetyl-CoA by pyruvate dehydrogenase and enters the Tricarboxylic Acid (TCA) cycle, generating NADH and FADH_2_, which fuel the mitochondrial electron transport chain and drive ATP synthesis (Ashcroft and Rorsman, 2013). Virtually all glucose entering the β-cell is directed toward mitochondrial oxidation. Minimal conversion of pyruvate to lactate is observed, which is explained by the suppressed expression of genes such as lactate dehydrogenase (LDHA) and the monocarboxylate transporter MCT1 (Slc16a1) (Maechler and Wollheim, 2001).

Increased mitochondrial oxidation leads to a rise in the intracellular ATP/ADP ratio. In β-cells, in addition to its energetic role, ATP is key in coupling glucose metabolism to electrical activity that leads to insulin secretion. The increase in the ATP/ADP ratio promotes the closure of ATP-sensitive potassium (K_ATP_) channels at the plasma membrane, resulting in membrane depolarization, and the opening of voltage-gated Ca^2+^ channels (VGCCs), allowing Ca^2+^ influx and an increase in [Ca^2+^]_i_ (Rizzuto et al., 2012).

Ca^2+^ activates the insulin vesicle fusion machinery by binding to sensor proteins such as synaptotagmin, which undergoes a conformational change and interacts efficiently with the SNARE complex. This complex promotes the fusion of secretory granules to the plasma membrane, ultimately leading to insulin exocytosis (Mu-u-min et al., 2023). In general, the increase in cytosolic Ca^2+^ results primarily from its influx through the plasma membrane, although mobilization from intracellular stores may also contribute to this signaling. Thus, the spatiotemporal regulation of intracellular Ca^2+^ dynamics constitutes a central element in the control of insulin secretion (Schulla et al., 2003).

## 3 Ca^2+^ dynamics in insulin secretion: channels, transporters and intracellular stores

### 3.1 Plasma membrane Ca^2+^ channels

Several studies demonstrate the importance of Ca^2+^ influx through plasma membrane channels for proper insulin release. Consistently, numerous studies show that inhibition of extracellular Ca^2+^ influx impairs GSIS (Schulla et al., 2003).

In general, β-cells possess multiple VGCCs distributed non-uniformly across the plasma membrane. This uneven distribution results in localized microdomains with high Ca^2+^ concentrations. L-type Ca^2+^ channels are the predominant VGCCs contributing to Ca^2+^ influx in β-cells and, consequently, to the triggering of insulin secretion. In mice, β-cell-specific deletion of CaV1.2 nearly abolished first-phase insulin secretion and caused glucose intolerance, indicating that this isoform plays a critical role in stimulus-secretion coupling (Schulla et al., 2003).

In addition to CaV1.2, CaV1.3 is also among the main L-type channels expressed in β-cells and contributes to Ca^2+^ entry associated with secretion. However, in rat INS-1 832/13 β-cells, for example, CaV1.2 silencing significantly reduced insulin secretion, whereas CaV1.3 silencing had no detectable effect, suggesting that CaV1.2 is the critical VGCC in this model (Nitert et al., 2008). It should be noted, however, that this cell line does not fully recapitulate the CaV isoform expression patterns observed in primary β-cells, which may influence the relative contribution of each channel. Consistent with this, CaV1.3 has been shown to be necessary for initiating glucose-induced electrical activity in murine β-cells and to modulate β-cell mass and insulin secretion in both young and aged mice (Namkung et al., 2001; Theiner et al., 2022). In humans, evidence indicates that CaV1.3 expression may be ten times higher than CaV1.2 in isolated β-cells (Reinbothe et al., 2013). Reduction of its expression impairs insulin secretion, and genetic variants of CaV1.3 are associated with T2D, suggesting its physiological relevance.

The Transient Receptor Potential Melastatin (TRPM) and Vanilloid (TRPV) channels also modulate Ca^2+^ entry and β-cell excitability, but rather than acting as primary depolarization-dependent triggers, they contribute to amplification and fine-tuning of insulin secretion.

TRPM2, which can be activated by adenosine dinucleotides, hydrogen peroxide, or intracellular Ca^2+^, has been shown to be involved in insulin secretion (Uchida and Tominaga, 2014). In rat pancreatic islets, elevated temperature (40 °C) increased GSIS, and this effect was reduced following TRPM2 silencing (Togashi et al., 2006). Additionally, TRPM2-deficient β-cells displayed reduced increases in [Ca^2+^]_i_ in response to glucose stimulation. Notably, this reduction did not differ between the early (0–10 min) and late (10–60 min) phases, suggesting that TRPM2 regulates overall intracellular Ca^2+^ levels independently of secretion phase. TRPM2 knockout (KO) mice also exhibited impaired glucose tolerance and insulin secretion, indicating that this channel integrates metabolic and hormonal signals (Uchida et al., 2011).

TRPM5 is another member of the TRPM subfamily, functioning as a Ca^2+^-activated monovalent channel that contributes to membrane depolarization and amplification of Ca^2+^ signaling supporting insulin secretion. TRPM5-deficient mice show reduced insulin secretion and glucose intolerance, and this channel has been implicated in regulating Ca^2+^ oscillation patterns and secretory amplification (Brixel et al., 2010). In rat islets, TRPM5 inhibition reduced insulin secretion induced by glucose, arginine, and GLP-1. However, studies in humans are limited, and TRPM5 expression appears relatively low compared to other TRPM channels such as TRPM4 and TRPM3 (Matos et al., 2022).

TRPV2 has also been implicated in GSIS. In murine models and mouse MIN6 β-cells, TRPV2 translocates from the cytoplasm to the plasma membrane in response to insulin and high glucose levels. This glucose-dependent recruitment enhances Ca^2+^ influx and potentiates insulin secretion, and inhibition or knockdown of TRPV2 reduces GSIS (Hisanaga et al., 2009).

Because insulin secretion depends on controlled oscillations in Ca^2+^ concentration, mechanisms responsible for Ca^2+^ removal are equally critical. Excess [Ca^2+^]_i_ can induce apoptosis, making Ca^2+^ extrusion essential (Orrenius et al., 2003). The primary systems involved are the plasma membrane Ca^2+^-ATPase (PMCA) and the Na^+^/Ca^2+^ exchanger (NCX). PMCA isoforms are abundantly expressed in β-cells (Hammes et al., 1996), supporting their role in Ca^2+^ extrusion and regulation of insulin secretion. Overexpression of PMCA2 in rat BRIN-BD11 β-cells significantly reduced cytosolic Ca^2+^ levels in response to glucose and decreased ER and mitochondrial Ca^2+^ levels (Jiang et al., 2010). Conversely, heterozygous deletion of PMCA2 increased intracellular Ca^2+^ stores, enhanced GSIS, and improved β-cell proliferation, mass, and viability (Pachera et al., 2015). In rat pancreatic β-cells, glucose exerted opposite effects on the two major Ca^2+^ extrusion systems, enhancing PMCA expression and activity while suppressing NCX expression and activity (Herchuelz et al., 2007). Heterozygous inactivation of NCX1 increased β-cell mass and proliferation and enhanced GSIS, highlighting its role in Ca^2+^ dynamics and its potential as a pharmacological target (Nguidjoe et al., 2011). Importantly, PMCA predominates at low to moderate Ca^2+^ levels due to its high affinity and low capacity, whereas NCX becomes more relevant at elevated Ca^2+^ concentrations due to its high capacity and low affinity (Brini and Carafoli, 2009).

Together, these systems constitute the core machinery governing Ca^2+^ dynamics across the plasma membrane in β-cells.

### 3.2 Intracellular Ca^2+^ stores and their channels

Two of the main intracellular Ca^2+^ stores are the ER and the mitochondria. In general, the ER exhibits both high capacity and high affinity for Ca^2+^, allowing it to sequester large amounts of this ion even at low cytosolic concentrations. In contrast, mitochondria are also highly specialized in accumulating large amounts of Ca^2+^ due to their high capacity; however, unlike the ER, they display low affinity for Ca^2+^ uptake, requiring higher local concentrations of this ion for mitochondrial Ca^2+^ entry (Rizzuto et al., 2012).

Ca^2+^ uptake into the ER occurs *via* the sarco/ER Ca^2+^-ATPase (SERCA), while Ca^2+^ release is mediated by inositol 1,4,5-trisphosphate receptors (IP_3_R) and ryanodine receptors (RyR) (Berridge et al., 2003). SERCA-mediated Ca^2+^ sequestration depends on cytosolic ATP levels, linking cellular energy status to Ca^2+^ homeostasis. This regulation maintains resting cytosolic Ca^2+^ concentrations between ∼25 and 100 nM and ER Ca^2+^ levels between ∼200 and 500 μM. SERCA2b and SERCA3 are the predominant isoforms in β-cells (Periasamy and Kalyanasundaram, 2007) and several studies have demonstrated their importance in pulsatile insulin secretion. Reduced SERCA2 expression or activity compromises ER Ca^2+^ storage and intracellular Ca^2+^ homeostasis, leading to β-cell dysfunction and impaired insulin secretion (Tong et al., 2016; Kono et al., 2012). Consistently, β-cell-specific SERCA2 deficiency reduces GSIS and promotes glucose intolerance (Iida et al., 2023).

ER Ca^2+^ release *via* IP_3_R occurs following activation of inositol 1,4,5-trisphosphate (IP_3_), generated through phospholipase C (PLC)-mediated cleavage of phosphatidylinositol 4,5-bisphosphate (PIP_2_) into IP_3_ and diacylglycerol (DAG). IP_3_ binds to its receptors at the ER membrane, promoting Ca^2+^ release into the cytosol (Clapham, 2007). Various stimuli can trigger IP_3_ production in β-cells, including activation of G protein-coupled receptors stimulated by extracellular nucleotides and free fatty acids. These receptors signal PLC activation, leading to IP_3_ generation and subsequent ER Ca^2+^ release *via* IP_3_R (Rorsman and Braun, 2013; Itoh et al., 2003). In general, intracellular Ca^2+^ mobilization mediated by the IP_3_R is essential for the formation of Ca^2+^ microdomains, as well as for the generation of coordinated oscillations that enable stimulus-secretion coupling during GSIS. Conversely, IP_3_R dysfunction can impair ER Ca^2+^ release, leading to altered Ca^2+^ signaling and reduced insulin secretion (Ehrlich, 1995).

ER Ca^2+^ release *via* RyR occurs through Ca^2+^-induced Ca^2+^ release (CICR), a positive feedback mechanism in which Ca^2+^ release promotes further RyR activation. Three RyR isoforms exist (RyR1, RyR2, and RyR3), with RyR2 being predominant in β-cells, although overall expression levels are relatively low. Despite this, RyRs exhibit high capacity for Ca^2+^ release, and several studies indicate their involvement in insulin secretion. Stimulation of human β-cells with ryanodine altered insulin secretion independently of glucose (Islam, 2002). Additional evidence shows that RyRs mediate CICR, amplifying cytosolic Ca^2+^ levels and contributing to fine-tuning Ca^2+^ signaling and insulin secretion. Moreover, RyR2 dysfunction in murine models and rare human mutations have been associated with glucose intolerance and impaired insulin secretion (Santulli et al., 2015).

Mitochondrial Ca^2+^ also plays a crucial role in linking energy metabolism to insulin secretion. Its dynamics are regulated through interactions between the ER, cytosol, and mitochondria. High-Ca^2+^ microdomains are formed at ER-mitochondria contact sites, known as mitochondria-associated membranes (MAMs) (Rizzuto et al., 1998), which enable efficient Ca^2+^ transfer between these organelles. In these regions, Ca^2+^ released from the ER *via* IP_3_R and RyR is rapidly taken up by mitochondria through the mitochondrial calcium uniporter (MCU). In this context, IP_3_R plays a central role in establishing Ca^2+^ microdomains at ER-mitochondria interfaces (Rizzuto et al., 1998; Csordás et al., 2018).

Although MCU has low affinity for Ca^2+^, it possesses high capacity, and the combination of high local Ca^2+^ concentrations and mitochondrial membrane potential enables efficient mitochondrial Ca^2+^ uptake (De Stefani et al., 2011). Within the mitochondrial matrix, Ca^2+^ enhances the activity of dehydrogenases involved in the TCA cycle, increasing NADH and FADH_2_ production and ultimately boosting ATP synthesis through oxidative phosphorylation (Denton, 2009; Vilas-Boas et al., 2023). This increase in the ATP/ADP ratio drives the downstream events leading to insulin secretion (Rizzuto et al., 1998).

Mitochondrial Ca^2+^ efflux occurs primarily *via* the Na^+^/Li^+^/Ca^2+^ exchanger (NCLX), and may also occur *via* a H^+^/Ca^2+^ exchanger under specific metabolic conditions, although this mechanism remains controversial. Additionally, Ca^2+^ can be released in a non-regulated manner under pathological conditions through the opening of the mitochondrial permeability transition pore (mPTP), typically triggered by Ca^2+^ overload within the mitochondrial matrix, oxidative stress, and cell death (Pathak and Trebak, 2018).

Numerous studies highlight the importance of mitochondrial Ca^2+^ dynamics in β-cell physiology, particularly in insulin secretion. In rat INS-1 832/13 β-cells and human EndoC-βH1 β-cells, silencing of MICU2, a regulatory component of the MCU complex, reduced mitochondrial Ca^2+^ uptake and impaired GSIS (Vishnu et al., 2021). Similarly, in rat INS-1E β-cells, MCU activation enhanced insulin secretion, whereas its inhibition impaired secretion (Bermont et al., 2020).

Importantly, not only mitochondrial Ca^2+^ uptake but also its efflux must be tightly regulated. In mouse MIN6 β-cells and primary β-cells (Nita et al., 2012), inhibition or deletion of NCLX lead to mitochondrial Ca^2+^ accumulation, reduced glucose-stimulated cytosolic Ca^2+^ increases, and impaired and delayed insulin secretion.

Collectively, these data point to the importance of coordinated ER and mitochondrial Ca^2+^ handling in shaping β-cell Ca^2+^ dynamics and insulin secretion.

### 3.3 Plasma membrane-ER coupling in Ca^2+^ signaling

Although it occurs under specific cellular conditions, Ca^2+^ dynamics must also account for store-operated Ca^2+^ entry (SOCE), a pathway that integrates the plasma membrane and the ER (Putney, 1990). In general, store-operated Ca^2+^ channels (SOCs) are voltage-independent and are activated by ER Ca^2+^ depletion, and are therefore associated with physiological or pathological conditions that disrupt Ca^2+^ homeostasis in this compartment.

SOCE is mediated by ER Ca^2+^ sensors STIM1/STIM2 and plasma membrane channels Orai1. The STIM1 or STIM2 proteins detect the reduction in ER luminal Ca^2+^ through their EF-hand domains; this decrease induces conformational changes, oligomerization, and migration of these proteins toward regions near the plasma membrane, where they interact with Orai1 channels. This interaction promotes the functional assembly of SOCs and allows extracellular Ca^2+^ influx into the cytosol *via* SOCE (Prakriya and Lewis, 2015).

In this context, it has been demonstrated that both STIM1 and Orai1 silencing in mouse MIN6 β-cells, as well as β-cell-specific deletion of the STIM1 gene, suppress SOCE and impair the potentiation of GSIS (Usui et al., 2019). Similarly, studies in rat INS-1E β-cells have shown that SOCE contributes to the regulation of GSIS, as pharmacological inhibition of SOC channels or Orai1 silencing reduces insulin secretion and abolishes the potentiating effect of acetylcholine following ER Ca^2+^ depletion (Sa et al., 2015).

In summary, the results regarding changes in GSIS resulting from modulation of intracellular Ca^2+^ homeostasis and dynamics (summarized in Table 1) indicate that intracellular Ca^2+^ homeostasis in β-cells does not depend on a single compartment but rather on an integrated network involving the ER, mitochondria, plasma membrane and cytosol. Key players, including SERCA, IP_3_R, RyR, MCU, NCLX, VGCC, TRPM, PMCA, NCX, and STIM/Orai, act in a coordinated manner to regulate both the amplitude and temporal dynamics of Ca^2+^ signaling. The ER functions as the main intracellular Ca^2+^ reservoir, controlling Ca^2+^ release and reuptake, while mitochondria act as metabolic sensors that convert Ca^2+^ signals into ATP production, enabling stimulus-secretion coupling. The formation of MAMs ensures efficient Ca^2+^ transfer between these organelles. Other intracellular microdomains, as plasma membrane-ER coupling may also play an important role. Disruptions in any of these components can impair Ca^2+^ signaling, energy metabolism, and ultimately insulin secretion, reinforcing the importance of inter-organelle communication in β-cell physiology.

**TABLE 1:** Modulation of Ca^2+^ dynamics and its impact on glucose-stimulated insulin secretion (GSIS). The table summarizes the key studies included in this review, highlighting the effects of genetic and chemical modulation of Ca^2+^ homeostasis on β-cell function and GSIS.

Model	Condition	Effect	References
Mouse pancreatic islets.	β-cell-specific deletion of the L-type Ca^2+^ channel CaV1.2.	Reduced GSIS, primarily due to impaired first-phase insulin secretion.	Schulla et al. (2003)
Rat INS-1 832/13 β-cell.	CaV1.2 or CaV1.3 siRNA-mediated silencing (72 h).	CaV1.2 knockdown significantly reduced GSIS, whereas CaV1.3 knockdown had no significant effect.	Nitert et al. (2008)
Mouse pancreatic islets.	Genetic deletion of the L-type Ca^2+^ channel α1D (CaV1.3).	No significant impairment of GSIS at stimulatory glucose concentrations (≥6 mM glucose), despite reduced insulin secretion at 3 mM glucose.	Namkung et al. (2001)
Mouse pancreatic islets.	Genetic deletion of CaV1.3.	Reduced sustained insulin release, associated with impaired glucose-induced electrical activity and decreased β-cell mass.	Theiner et al. (2022)
• Human pancreatic islets. • Rat INS-1 832/13 β-cells.	CACNA1D (CaV1.3) siRNA-mediated silencing (48 h).	Reduced GSIS and impaired exocytotic activity.	Reinbothe et al. (2013)
• Rat pancreatic islets. • Rat RIN-5F β-cells.	TRPM2 siRNA-mediated silencing (48–72 h).	Significantly reduced GSIS; cADPR-mediated TRPM2 activation at physiological temperature contributes to insulin secretion.	Togashi et al. (2006)
Mouse pancreatic islets.	Global genetic deletion of TRPM2 (constitutive knockout model).	Reduced GSIS, affecting both the first and second phases of insulin secretion.	Uchida et al. (2011)
Mouse pancreatic islets.	Global genetic deletion of TRPM5 (constitutive knockout model).	Reduced GSIS, particularly at elevated glucose concentrations.	Brixel et al. (2010)
• Mouse MIN6 β-cells. • Mouse pancreatic islets.	TRPV2 siRNA-mediated silencing (48 h); TRPV2 inhibition with tranilast (100 μM).	Reduced Ca^2+^ influx and impaired GSIS.	Hisanaga et al. (2009)
Mouse pancreatic islets.	Heterozygous genetic deletion of PMCA2 (PMCA2^+/−^).	Increased ER Ca^2+^ stores and enhanced biphasic GSIS.	Pachera et al. (2015)
Mouse pancreatic islets.	Heterozygous genetic deletion of NCX1 (NCX1^+/−^).	Reduced Ca^2+^ extrusion increased intracellular and ER Ca^2+^ levels, enhancing biphasic GSIS.	Nguidjoe et al. (2011)
Mouse pancreatic islets.	β-cell-specific deletion of SERCA2 with high-fat diet (60% kcal from fat, 25 weeks).	Reduced ER Ca^2+^ homeostasis impaired glucose-induced Ca^2+^ signaling and GSIS.	Tong et al. (2016)
Rat INS-1 cells.	Hyperglycemic and cytokine stress-induced SERCA2 downregulation. Restoration of SERCA2 expression by PPAR-γ activation with pioglitazone (10 μM, 24 h).	GSIS was impaired under hyperglycemic and cytokine stress and restored following pioglitazone treatment.	Kono et al. (2012)
Mice carrying RyR2 mutations associated with Catecholaminergic Polymorphic Ventricular Tachycardia (RyR2-R2474S or RyR2-N2386I).	Chronic ER Ca^2+^ leak through RyR2.	Reduced GSIS and impaired glucose homeostasis.	Santulli et al. (2015)
• Rat INS-1 832/13 β-cells. • Human EndoC-βH1 cells. • Mouse pancreatic islets.	MICU2 siRNA-mediated silencing (48–72 h); global genetic deletion of MICU2 (Micu2^−/−^).	Reduced GSIS associated with impaired mitochondrial Ca^2+^ uptake.	Vishnu et al. (2021)
• Rat INS-1E β-cells. • Human pancreatic islets.	MCU activation with kaempferol (10 μM, 30 min); MCU inhibition with mitoxantrone (10 μM, 30 min).	MCU activation with kaempferol (10 μM, 30 min); MCU inhibition with mitoxantrone (10 μM, 30 min).	Bermont et al. (2020)
• Mouse MIN6 β-cells. • Primary mouse β-cells.	NCLX siRNA-mediated silencing; dominant-negative NCLX expression (dnNCLX).	Impaired GSIS, characterized by a delayed insulin secretory response and altered glucose-dependent Ca^2+^ signaling.	Nita et al. (2012)
• Mouse MIN6 β-cells. • Mouse pancreatic islets.	GPR40 activation with fasiglifam (TAK-875, 5 μM) STIM1 and Orai1 silencing β-cell-specific deletion of STIM1.	Fasiglifam Enhanced GSIS following GPR40 activation; this response was attenuated by STIM1/Orai1 silencing or β-cell-specific STIM1 deletion.	Usui et al. (2019)
• Rat INS-1E cells. • Rat pancreatic islets.	Orai1 or TRPC1 siRNA-mediated silencing (72 h); SOCE inhibition with YM-58483 (1 μM).	Reduced GSIS following Orai1 or TRPC1 silencing and after pharmacological inhibition of SOCE.	Sa et al. (2015)

## 4 Reactive oxygen species (ROS) and their main sources in β-cells

Reactive oxygen species (ROS), generated during both normal and altered metabolism (Sies and Jones, 2020), are also key signaling molecules in GSIS. They arise from the incomplete reduction of molecular oxygen through sequential one-electron transfers. Upon accepting one electron, molecular oxygen forms the superoxide anion radical (O_2_^•-^) (Equation 1), which can be protonated to generate the hydroperoxyl radical (HO_2_^•^) (Equation 2), or undergo dismutation/reduction, in a reaction catalyzed by SOD, to form hydrogen peroxide (H_2_O_2_) (Equation 3). H_2_O_2_, in turn, can participate in further reactions, such as the Fenton reaction, leading to the formation of the hydroxyl radical (^•^OH) (Equation 4), which, upon reduction, ultimately results in the formation of water (H_2_O) (Equation 5) (Sies and Jones, 2020; Halliwell and Gutteridge, 2015).

$$O_{2}+e^{-}\to \;O_{2}\cdot^{-}$$ (1)

$$O_{2}\cdot^{-}+H^{+}⇌\;HO_{2}\cdot$$ (2)

$$2\;O_{2}\cdot^{-}+2\;H^{+}\to \;H_{2}O_{2}+O_{2}$$ (3)

$$H_{2}O_{2}+Fe^{2+}\to \;\cdot OH+OH^{-}+Fe^{3+}$$ (4)

$$\cdot OH+e^{-}+H^{+}\to \;H_{2}O$$ (5)

Many ROS are generated by the mitochondrial electron transport chain, as byproducts, by NADPH oxidase (NOX) enzymes, the ER during oxidative protein folding, peroxisomes and other oxidases, such as xanthine oxidase, monoamine oxidase, and cytochrome P450 (Finkel, 2011).

Among ROS, superoxide shows limited direct reactivity, acting mainly as a precursor of other species, including H_2_O_2_ and peroxynitrite (Swartz et al., 2005). In contrast, H_2_O_2_ is more stable, diffusible, and functions as a selective second messenger, with its transport facilitated by specialized aquaporins (AQP) known as peroxiporins, as AQP8 in β-cells (Bienert et al., 2007; Krüger et al., 2021). While ROS play physiological roles in redox signaling, metabolism, and proliferation, excessive or sustained production disrupts redox balance, causing oxidative stress and biomolecular damage (Schieber and Chandel, 2014).

In β-cells, tight regulation of ROS production and removal is essential for proper function, and its disruption is linked to mitochondrial dysfunction and diabetes *mellitus* (DM) (Dinić et al., 2022). In this context, mitochondria and NOXs are the main ROS sources.

The O_2_^•-^ released into the mitochondrial matrix is converted into H_2_O_2_ *via* a dismutation reaction catalyzed by superoxide dismutase 2 (SOD2), whereas O_2_^•-^ released into the intermembrane space or any O_2_^•-^ diffusing into the cytosol is dismutated by SOD1 (Murphy, 2009). H_2_O_2_ is continuously degraded by catalase and glutathione peroxidases, in which it is reduced to water (Winterbourn, 1995; Sies, 2017).

NOXs comprise a family of multiprotein enzymatic complexes whose primary function is ROS generation, in contrast to mitochondria where ROS are byproducts (Babior, 1999; Bedard and Krause, 2007). Currently, seven NOX isoforms have been described, which are differentially distributed according to tissue specificity and distinct regulatory mechanisms of ROS production. These enzymes produce either O_2_^•-^ or H_2_O_2_, contributing to spatially restricted redox signaling (Sies, 2017), as ROS signaling is likely to be more relevant near their site of production, given their reactivity and rapid removal by antioxidant systems (Finkel, 2011). In this sense, NOX intracellular localization is of great interest and will be discussed later in this review.

Similarly to mitochondrial oxidants, those generated by NOXs play a dual role. Under physiological conditions, they participate in the regulation of insulin secretion and cellular signaling. However, their excessive or sustained activation contributes to cellular dysfunction. Thus, proper redox balance, rather than absolute ROS levels, is critical for maintaining insulin secretion and β-cell physiology.

### 4.1 Redox signaling in insulin secretion

ROS-mediated signaling plays an important role in GSIS and occurs mainly through the modification of redox-sensitive proteins (Pi et al., 2010). Among ROS, H_2_O_2_ is mostly important due to its relative stability and ability to act as a second messenger. It acts through reversible oxidation of cysteine residues, forming sulfenic acid (-SOH) in enzymes, ion channels, and signaling proteins. It is known that K_ATP_ channels and VGCC are redox-sensitive and key redox targets in β-cells. Oxidation of cysteine residues (-SH) in the regulatory subunit (SUR1) of K_ATP_ enhances channel closure, reinforcing ATP-dependent inhibition, which promotes membrane depolarization and subsequent Ca^2+^ influx, amplifying insulin secretion (Ježek et al., 2021). Importantly, these effects occur within spatially restricted redox microdomains, where H_2_O_2_ can selectively modify nearby targets due to its limited diffusion and local reactivity.

Several evidence support a physiological role for mitochondria and NOX-derived ROS in GSIS and the main findings are summarized in Table 2. Pharmacological inhibition of NOX or silencing of one of its subunits reduces GSIS in rat islets (Morgan et al., 2009), and NOX activity is involved in palmitate-induced increases in superoxide and insulin secretion (Graciano et al., 2011). In human islets, NOX2 inhibition compromises glucose metabolism and GSIS (Rebelato et al., 2012) and NOX4, which constitutively produces H_2_O_2_ in association with glucose metabolism, has emerged as a key regulator of GSIS, as its inhibition or deletion impairs insulin secretion (Plecitá-Hlavatá et al., 2020). Additionally, there is evidence that mitochondria-derived ROS are required for GSIS (Leloup et al., 2009).

**TABLE 2:** Effects of ROS homeostasis modulation on glucose-stimulated insulin secretion (GSIS). The table summarizes the key studies included in this review, highlighting the effects of genetic and chemical interventions targeting ROS production, scavenging, and antioxidant on β-cell function and GSIS.

Model	Condition	Effect	References
Rat pancreatic islets.	NOX inhibition with VAS2870 (10 μM); antioxidant treatment with PEG-catalase (500 U/mL).	Reduced GSIS, indicating that NOX-derived H_2_O_2_ contributes to glucose-stimulated insulin secretion.	Morgan et al. (2009)
Rat pancreatic islets.	Palmitate treatment (1 mM, 1 h); NOX inhibition with VAS2870 (10 μM).	Palmitate enhanced insulin secretion, whereas NOX inhibition attenuated the palmitate-induced secretory response.	Graciano et al. (2011)
Human pancreatic islets.	NOX2 inhibition with DPI or apocynin.	Impaired GSIS associated with reduced glucose oxidation following NOX2 inhibition.	Rebelato et al. (2012)
• Mouse pancreatic islets. • Rat INS-1E β-cells.	NOX4 deficiency/inhibition (reduced cytosolic H_2_O_2_ signaling).	Reduced GSIS; first-phase insulin secretion abolished, indicating that NOX4-derived H_2_O_2_ is required for glucose-stimulated insulin secretion.	Plecitá-Hlavatá et al. (2020)
Rat pancreatic islets.	ROS scavenging with Trolox (antioxidant).	Reduced GSIS following attenuation of mitochondrial ROS signaling.	Leloup et al. (2009)
Rat pancreatic islets.	Fasting for 48 h; NOX inhibition with VAS2870 (20 μM) or DPI (5 μM).	Reduced GSIS following ROS scavenging or NOX inhibition, supporting a role for ROS signaling in glucose-induced insulin secretion.	Munhoz et al. (2016)
Mouse MIN6 β-cells.	γ-Glutamylcysteine synthetase (γ-GCS) knockdown by ribozyme transfection (stable transfection), resulting in reduced intracellular glutathione (GSH) levels.	Enhanced insulin secretion in response to glucose (10 mM glucose).	Kondo et al. (2000)
• Mouse pancreatic islets. • Rat INS-1 (832/13) β-cells.	H_2_O_2_ scavenging with cell-permeable catalase or N-acetyl-L-cysteine. (NAC); H_2_O_2_ elevation with exogenous H_2_O_2_ or diethyl maleate.	Reduced H_2_O_2_ accumulation inhibited GSIS, whereas increased intracellular H_2_O_2_ stimulated insulin secretion, supporting a signaling role for H_2_O_2_ in GSIS.	Pi et al. (2007)

However, the effects of ROS are highly context-dependent. Under low-glucose conditions, NOX-derived ROS may contribute to the suppression of insulin secretion, suggesting a protective role of NOX against hypoglycemia, whereas under high-glucose, increased NADPH production enhances antioxidant system activity, leading to greater ROS clearance and allowing insulin secretion to occur (Munhoz et al., 2016). Importantly, chronic exposure to glucolipotoxicity and/or inflammation leads to sustained ROS production, oxidative stress and β-cell dysfunction, as will be discussed in further sections.

The balance between ROS generation and antioxidant capacity is therefore critical. Modulation of endogenous antioxidant systems directly impacts insulin secretion. For instance, reduced glutathione (GSH) synthesis increases insulin secretion in mouse MIN6 β-cells (Kondo et al., 2000) and depletion of intracellular GSH or exposure to exogenous H_2_O_2_ enhances GSIS in β-cell models, whereas high antioxidant levels suppress it (Pi et al., 2010). Importantly, ROS alone are insufficient to drive insulin secretion: inhibition of the mitochondrial respiratory chain increases ROS production but reduces ATP synthesis, resulting in impaired GSIS, highlighting the importance of metabolic coupling (Pi et al., 2007).

β-cells exhibit a remarkable redox profile. While glucose enhances SOD1 activity in rat islets (Oliveira et al., 1999), β-cells express relatively low levels of catalase and glutathione peroxidase compared to other tissues (Lenzen, 2008; Lenzen et al., 1996). This observation historically supported the view that β-cells are more susceptible to oxidative damage. However, this perspective has been refined by more recent evidence.

In this sense, β-cells exhibit highly efficient antioxidant and redox-regulated systems, such as glutaredoxins, thioredoxins, and peroxiredoxins (Benáková et al., 2021; Stancill and Corbett, 2023), which not only detoxify H_2_O_2_ but also function as redox sensors and signaling mediators. Peroxiredoxins, in particular, play a central role by transferring oxidative equivalents to target proteins through redox relay mechanisms within confined cellular regions, modulating signaling pathways, while thioredoxins restore their reduced state (Rhee and Woo).

This organization allows β-cells to effectively cope with transient and physiological ROS fluctuations and reinforces the importance of H_2_O_2_ in redox signaling in this cell type. In line with this, glucose induces changes in the redox environment in β-cells. Increased glucose availability promotes reversible oxidation of cysteine residues in proteins involved in glycolysis, the TCA cycle, oxidative phosphorylation, ER protein folding, and insulin secretion. Under glucotoxic conditions (e.g., 25 mM glucose for 24 h), increased ROS production and oxidative protein modifications are observed without compromising cell viability, suggesting an adaptive response rather than immediate toxicity (Holendová et al., 2024a). This is accompanied by upregulation of antioxidant systems, including SODs and the peroxiredoxin/thioredoxin system, as well as increased oxidation of proteins such as PDI and ERO1, which are involved in insulin biosynthesis (Holendová et al., 2024a; Sun et al., 2015).

However, although their peroxiredoxin/thioredoxin system is robust, β-cells appear to have limited capacity to further upregulate antioxidant defenses under conditions of chronic metabolic or inflammatory stress. As a result, they appear to be relatively well adapted to acute and reversible redox signaling, but become progressively susceptible to sustained oxidative stress, as observed in T2D.

Thus, instead of being intrinsically fragile, β-cells display a finely tuned redox balance that supports signaling under physiological conditions. In this context, controlled ROS generation appears to be essential for β-cell physiology, particularly for GSIS, while its chronic dysregulation contributes to β-cell dysfunction.

Importantly, the transition from physiological redox signaling to pathological oxidative stress is not defined by ROS levels alone, but rather by the interplay of several factors combined. The duration and amplitude of ROS exposure and the site-specific ROS production are of great importance, as transient and spatially restricted ROS signals support GSIS, while sustained or excessive ROS promotes cumulative damage. Additionally, β-cell antioxidant capacity also plays an important role in buffering ROS fluctuations, being largely dependent on NADPH availability and the efficiency of systems such as peroxiredoxin/thioredoxin and glutathione. The antioxidant response is not homogeneous across the cell. Instead, local NADPH availability, glutathione- and thioredoxin-dependent systems, defines the redox buffering capacity within specific subcellular compartments, such as the cytosol, mitochondria, and ER (Roma and Jonas, 2020). Thus, ROS signaling is determined by the interplay between site-specific ROS generation and the efficiency of antioxidant defenses operating within the same microenvironment. Finally, the metabolic and inflammatory context strongly modulates this balance, as chronic exposure to hyperglycemia, elevated fatty acids, and pro-inflammatory cytokines increases ROS generation and impairs antioxidant defenses. All these factors define a dynamic balance that determines whether ROS act as signaling molecules in GSIS or mediators of β-cell dysfunction.

### 4.2 Intracellular localization and compartmentalized signaling of NOXs

Several lines of evidence in non-β-cells support the concept that different NOX isoforms exhibit different intracellular localization and thus operate within spatially defined redox microdomains. At the plasma membrane, NOX1 and NOX2 are well-established sources of extracellular ROS involved in receptor and ion channel modulation (Bedard and Krause, 2007; Brown and Griendling, 2009). Furthermore, NOX2 has been shown to assemble within lipid rafts and specialized membrane microdomains, forming redox signaling platforms that enable highly localized regulation of target proteins (Ushio-Fukai, 2009; Nordzieke and Medraño-Fernandez, 2018). NOX4 is consistently associated with the ER, where it contributes to luminal redox regulation and ER stress signaling (Sciarretta et al., 2013; Laurindo et al., 2014). NOX2 and, in some contexts, NOX4 have been localized to intracellular vesicles and endosomal compartments, supporting roles in localized ROS generation within trafficking pathways (Bedard and Krause, 2007).

In pancreatic β-cells, however, the intracellular localization of NOX isoforms remains less well characterized. To address this, our group recently investigated their subcellular distribution in rat INS-1E β-cells (De Almeida et al., 2025). We found that NOX1 and DUOX1 are enriched in the ER, DUOX2 localizes to insulin-containing vesicles, and NOX2 and NOX4 are found across vesicles, ER, and the plasma membrane. This organization enables selective redox modulation of nearby targets rather than global oxidative effects and may be critical for integrating redox signaling with Ca^2+^ dynamics and stimulus-secretion coupling.

At the ER, NOX activity may contribute to the regulation of the luminal redox environment required for protein folding, but excessive ROS may promote ER stress and disrupt Ca^2+^ handling through redox-sensitive targets such as IP_3_R, RyR, and SERCA pump. In vesicular compartments, NOX enzymes may influence insulin processing and exocytosis by modulating the redox state of granule-associated proteins. At the plasma membrane, NOX-derived H_2_O_2_ can regulate ion channels such as K_ATP_ and VGCC, linking metabolic signals to membrane depolarization and Ca^2+^ influx.

In addition to spatial organization, NOX enzymes are dynamically regulated under stress conditions. Our group has also demonstrated that pro-inflammatory cytokines induce a temporally coordinated remodeling of NOX expression, suggesting a shift from controlled redox signaling to more sustained oxidative conditions during β-cell stress (De Almeida et al., 2025). Importantly, NOX enzymes actively participate in stimulus-secretion coupling. NOX4, in particular, has been identified as a significant source of H_2_O_2_ that is required for proper GSIS (Plecitá-Hlavatá et al., 2020). Overall, compartmentalized NOX activity emerges as a central mechanism for the precise control of redox signaling in β-cells.

Despite this emerging model of compartmentalized and functionally specialized NOX signaling, many key aspects remain to be resolved. In particular, the relative contribution of individual NOX isoforms is still unclear, as many studies rely on pharmacological inhibitors with limited specificity and most mechanistic insights derive from β-cell lines or rodent islets, which may not fully recapitulate human β-cell physiology. While NOX4 has been proposed as a constitutive source of signaling-relevant H_2_O_2_, and NOX1/NOX2 as inducible sources under stress conditions, it is not yet known how exactly these roles depend on the context. Overall, current evidence supports a model in which NOX enzymes act less as primary generators of oxidative stress and more as spatially and temporally regulated amplifiers of redox signaling within defined subcellular microdomains.

## 5 MAMs as platforms for Ca^2+^-ROS crosstalk

Several proteins mediate ER-mitochondria communication at MAMs. Grp75 facilitates Ca^2+^ transfer through the VDAC1-IP_3_R1-Grp75 complex, modulating VDAC1 properties (Szabad et al., 2006), and promoting proliferation, while protecting against Ca^2+^-induced cell death when overexpressed (Wadhwa et al., 2002). Co-immunoprecipitation assays indicate that Grp75 associates with both IP_3_R and VDAC, linking these proteins without direct interaction (Szabad et al., 2006). IP_3_R also enhances mitochondrial Ca^2+^ uptake, supporting its role within a MAM-localized complex that regulates Ca^2+^ transfer to mitochondria.

Proteins involved in mitochondrial dynamics also contribute to MAM organization (De Brito and Scorrano, 2008). MFN2 acts as a physical tether between the ER and mitochondria, and its silencing increases organelle distance and impairs Ca^2+^ transfer in HeLa cells and MEFs. Interactions between ER-localized MFN2 and mitochondrial MFN1/MFN2 promote close contacts and formation of Ca^2+^ microdomains, enabling efficient Ca^2+^ transfer from IP_3_R to mitochondria *via* MCU. Thus, both Grp75 and MFN2 are critical for maintaining effective ER-mitochondria Ca^2+^ communication.

MAMs are also sites of intense Ca^2+^-ROS crosstalk. IP_3_R and VDAC are redox-sensitive proteins whose cysteine residues undergo oxidative modifications. In IP_3_R, moderate oxidation enhances channel activity, whereas excessive oxidation impairs function and disrupts Ca^2+^ homeostasis (Joseph et al., 2018). Consistently, oxidizing agents and NOX-derived O_2_^•-^ increase IP_3_R-mediated Ca^2+^ signaling (Joseph et al., 2018; Bánsághi et al., 2014), and mitochondrial ROS support IP_3_R-dependent Ca^2+^ oscillations (Booth et al., 2016). VDAC is similarly modulated by ROS, with H_2_O_2_-induced cysteine oxidation influencing channel conductance and gating (Reina et al., 2020), potentially impacting Ca^2+^ transfer through the IP_3_R–Grp75–VDAC complex. Although these studies were not performed in pancreatic β-cells, they reinforce the relevance of Ca^2+^-ROS crosstalk in regulating intracellular Ca^2+^ signaling.

Overall, MAMs function as key hubs integrating Ca^2+^ and ROS signaling (Figure 3). In pancreatic β-cells, which rely on Ca^2+^ for insulin secretion (Rorsman and Renstrm, 2003) and possess a specialized antioxidant system (Ježek et al., 2021), alterations in MAM-associated proteins (e.g., Grp75, MFN2, IP_3_R, VDAC) may disrupt ER-mitochondria communication and metabolism-secretion coupling (Vig et al., 2021; Madec et al., 2021). Redox-dependent modulation of Ca^2+^ transfer may support physiological homeostasis or, under stress conditions, contribute to mitochondrial dysfunction, β-cell failure, and diabetes development.

## 6 Obesity and T2D: role of Ca^2+^ and ROS

T2D typically develops in the context of obesity and metabolic syndrome and is a progressive condition characterized by an increase in insulin resistance (IR) in peripheral tissues.

Obesity is associated with elevated circulating fatty acids (Abdul-Ghani et al., 2008; Hershkop et al., 2016; Gastaldelli et al., 2017) and white adipose tissue remodeling, characterized by adipocyte hypertrophy and increased release of free fatty acids (FFAs) and pro-inflammatory cytokines, which contribute to low-grade systemic inflammation (Blüher, 2013; Hotamisligil, 2017). Consequently, glucose uptake in skeletal muscle and white adipose tissue is impaired, while hepatic glucose production increases, resulting in hyperglycemia (Vilas-Boas et al., 2021a). Increased FFA flux further promotes ectopic lipid accumulation and metabolic dysfunction.

Hyperglycemia, together with lipotoxicity and inflammation associated with chronic obesity, lead to an increase in insulin demand as a consequence of IR. Initially, pancreatic β-cells attempt to compensate for IR by enhancing insulin synthesis and secretion; however, at later stages, they undergo decompensation, characterized by a decline in β-cell function and mass, leading to the development of T2D (Vilas-Boas et al., 2021a; DeF and ronzo, 2009).

These processes have been extensively studied in animal models and the main findings are summarized in Table 3. It has been shown that female mice fed a high fat diet (HFD) for 12 weeks and ob/ob mice develop obesity, IR, hyperinsulinemia, increased β-cell mass, and enhanced glucose-induced Ca^2+^ signals and GSIS (Gonzalez et al., 2013; Irles et al., 2015). Similarly, western diet-fed mice show early compensatory Ca^2+^ dynamics and insulin secretion, although prolonged exposure leads to a sex-dependent β-cell dysfunction (Visa and Berggren, 2024).

**TABLE 3:** Alterations in Ca^2+^ homeostasis associated with impaired glucose-stimulated insulin secretion (GSIS) under diabetogenic conditions. Summary of studies investigating how diabetogenic conditions, including glucotoxicity, lipotoxicity, obesity, and inflammation, alter Ca^2+^ signaling and contribute to impaired GSIS in pancreatic β-cells.

Model	Condition	Effect	References
Mouse pancreatic islets.	High-fat diet-induced obesity and hyperinsulinemia (60% kcal from fat, 12 weeks).	Enhanced GSIS, associated with increased glucose-induced Ca^2+^ signaling in β-cells.	Gonzalez et al. (2013)
Mouse pancreatic islets.	Genetic obesity and prediabetes (female New Zealand Obese mice).	Enhanced GSIS, associated with increased glucose-induced Ca^2+^ signaling and amplified intracellular signaling responses in β-cells.	Irles et al. (2015)
• Mouse pancreatic islets. • Human pancreatic islets.	High-fat diet feeding (mice) or glucolipotoxic conditions (human islets).	Enhanced β-cell Ca^2+^ coordination increased compensatory insulin secretion, whereas loss of Ca^2+^ coordination preceded β-cell dysfunction.	Visa and Berggren (2024)
Rat pancreatic islets.	Metabolic syndrome induced by a sucrose-rich diet (20% sucrose in drinking water, 8 weeks).	Enhanced GSIS, associated with increased voltage-dependent Ca^2+^ currents and altered K_ATP_ channel activity in β-cells.	Velasco et al. (2012)
Mouse pancreatic islets.	High-fat diet feeding for 15 weeks.	Reduced GSIS, associated with impaired coupling between voltage-gated Ca^2+^ channels and insulin secretory vesicles despite preserved global Ca^2+^ influx.	Collins et al. (2010)
Rat pancreatic islets.	Aging (24-month-old vs. 3-month-old rats).	Reduced GSIS, associated with impaired glucose-induced Ca^2+^ handling and diminished intracellular Ca^2+^ responses in β-cells.	Ribeiro et al. (2012)
Mouse pancreatic islets.	Low-dose cytokine treatment (TNF-α, IL-1β, and IFN-γ).	Reduced GSIS, associated with impaired glucose-induced Ca^2+^ handling and disrupted Ca^2+^ oscillatory activity.	Dula et al. (2010)
• Mouse MIN6 β-cells. • Human pancreatic β-cells.	Palmitate treatment (0.5 mM, acute and chronic exposure).	Impaired GSIS, associated with ER Ca^2+^ depletion, elevated cytosolic Ca^2+^ levels, and disrupted intracellular Ca^2+^ homeostasis.	Gwiazda et al. (2009)
• MIN6 mouse β-cells. • Mouse pancreatic islets.	Palmitate-induced lipotoxicity (0.5 mM PA) ± nifedipine or diazoxide.	Palmitate impaired GSIS; inhibition of Ca^2+^ influx with nifedipine or diazoxide partially restored GSIS and reduced ER stress.	Zhou et al. (2015b)
Mouse pancreatic islets.	β-cell-specific deletion of calcineurin B1 (Cnb1), disrupting. Ca^2+^/calcineurin-NFAT signaling.	Reduced β-cell mass and pancreatic insulin content, leading to hypoinsulinemia and age-dependent diabetes.	Heit et al. (2006)
Mouse pancreatic islets.	Genetic ablation of SERCA3 (SERCA3^−/−^).	Enhanced GSIS at high glucose despite normal glucose homeostasis *in vivo*, associated with altered intracellular Ca^2+^ oscillations.	Arredouani et al. (2002)
Human pancreatic islets from donors with type 2 diabetes.	Type 2 diabetes-associated mitochondrial dysfunction.	Reduced GSIS, associated with mitochondrial abnormalities, impaired oxidative metabolism, and increased ROS production.	Anello et al. (2005)
Mouse pancreatic islets from aged mice (18–24 months).	Aging-associated mitochondrial dysfunction with reduced K_ATP_ channel activity.	GSIS was maintained despite mitochondrial deficiency, associated with increased β-cell excitability and enhanced Ca^2+^ influx secondary to reduced K_ATP_ channel activity.	Gregg et al. (2016)
• Rat INS-1E β-cells. • Human pancreatic islets.	Acute (15 mM) or chronic glucotoxic glucose exposure (30 mM, 96 h).	Acute glucose stimulation enhanced GSIS, associated with increased ER-to-mitochondria Ca^2+^ transfer. Chronic glucotoxicity impaired GSIS, associated with disrupted ER-mitochondria Ca^2+^ exchange and altered mitochondrial Ca^2+^ handling.	Dingreville et al. (2019)
• Mouse pancreatic islets. • Mouse MIN6 β-cells.	SERCA activation with CDN1163 (10–30 μM); palmitate-induced lipotoxicity (0.5 mM).	SERCA activation enhanced GSIS and glucose-induced Ca^2+^ signaling. CDN1163 prevented palmitate-induced ER Ca^2+^ depletion, mitochondrial dysfunction, and GSIS impairment.	Nguyen et al. (2023)
• Human pancreatic islets from donors with type 2 diabetes. • Mouse MIN6 β-cells treated with palmitate.	Type 2 diabetes-associated reduction of ER–mitochondria contacts (MAMs).	Reduced GSIS, associated with decreased ER-to-mitochondria Ca^2+^ transfer and impaired organelle communication.	Thivolet et al. (2017)

However, this compensatory state is not sustained. Despite the observed compensatory upregulation, impaired coordination of Ca^2+^ signals between individual β-cells from ob/ob islets is observed, suggesting a lack of synchrony as an early functional defect that may contribute to β-cell failure during diabetes progression. In line with this, studies in metabolic syndrome models show that, although β-cells exhibit enhanced insulin secretion associated with altered K_ATP_ channel sensitivity and increased variability in Ca^2+^ currents, a subset of cells displays reduced Ca^2+^ currents, suggesting the emergence of functionally exhausted β-cell subpopulations under prolonged metabolic stress (Velasco et al., 2012). Other studies have shown that HFD-fed mice present loss of rapid exocytosis linked to a dispersion of Ca^2+^ entry, reflecting a functional dissociation between voltage-gated Ca^2+^ influx and the exocytotic machinery (Collins et al., 2010).

In addition to dysfunction in Ca^2+^ signaling, oxidative stress is also a prominent feature of obesity and T2D (Cecerska-Heryć et al., 2025). HFD-fed rat show hyperglycemia and impaired glucose tolerance, elevated ROS levels and decreased insulin content in isolated islets (Yuan et al., 2010a). Islets from Wistar rats intravenously infused with high glucose exhibit decreased GSIS and elevated total and mitochondrial ROS levels (Tang et al., 2007), in addition to activation of ER stress (Tang et al., 2012). In serum from obese ob/ob mice and human T2D islets, oxidative stress markers are increased (Zhou S. et al., 2015; MacDonald et al., 2013). Consistent with this, NOX4-derived ROS have been shown to contribute to inflammasome activation and local islet inflammation under nutrient overload (Holendová et al., 2024b).

Exposure of human/rodent islets and β-cell lines to glucolipotoxic and/or inflammatory conditions–commonly modeled *in vitro* and *ex vivo* using high glucose, fatty acids and/or inflammatory cytokines–to mimic the metabolic environment of obesity and T2D, leads to β-cell dysfunction and failure. These effects are driven by multiple stress pathways, such as oxidative stress and disruption of Ca^2+^ signaling. In the next sections, we will explore these aspects.

## 7 Ca^2+^ and T2D

As Ca^2+^ is a key intracellular second messenger in GSIS, alterations in its dynamics may contribute to β-cell dysfunction in conditions of obesity, prediabetes and T2D.

Evidence from human and animal studies supports an early disruption of Ca^2+^ handling in β-cell dysfunction. A study in human islets shows that genes involved in insulin secretion and Ca^2+^-dependent exocytosis are downregulated in T2D (Li et al., 2018), while aging is associated with lower [Ca^2+^]_i_ and impaired GSIS in rat islets (Ribeiro et al., 2012). Notably, this study indicates that β-cell dysfunction is already present during aging, even before the onset of overt T2D, and is closely associated with disrupted Ca^2+^ handling.

In early T2D models, isolated mouse islets exhibit elevated [Ca^2+^]_i_ at a low glucose concentration and disrupted [Ca^2+^]_i_ oscillations in response to high glucose, disturbances attributed to greater Ca^2+^ influx through L-type Ca^2+^ channels and reduced ER Ca^2+^ storage (Dula et al., 2010). In line with this, exposure of rat INS-1 832/13 β-cells to high glucose and inflammatory cytokines leads to a loss of SERCA2b expression, resulting in increased cytosolic Ca^2+^, impaired insulin secretion, and cell death (Kono et al., 2012).

Besides glucotoxic and inflammatory stress, lipotoxicity is another major contributor to β-cell dysfunction. Exposure of β-cells to FFAs, especially saturated ones, also have detrimental effects on Ca^2+^ signaling. In human islets and mouse MIN6 β-cells, palmitate (PA) rapidly elevates cytosolic Ca^2+^ *via* ER Ca^2+^ depletion and extracellular Ca^2+^ influx, causing ER stress, and β-cell death (Gwiazda et al., 2009). However, the long-lasting Ca^2+^ oscillations are poorly coupled to insulin exocytosis (Gwiazda et al., 2009). Pharmacological inhibition of PA-induced Ca^2+^ influx with nifedipine (L-type Ca^2+^ channel blocker) or diazoxide (K_ATP_ activator) attenuates ER stress, reduces apoptosis, and partially restores insulin secretion (Zhou Y. et al., 2015). Unsaturated FFAs such as linoleic acid also alter Ca^2+^ dynamics in rat islets, by inducing Ca^2+^ release from intracellular stores, and promoting Ca^2+^-dependent inactivation of VGCCs (Feng et al., 2008).

Ca^2+^ signaling also involves other downstream pathways and exocytotic machinery. Both insufficient and excessive activation of Ca^2+^-dependent pathways, such as ERK and calcineurin, impair β-cell function, highlighting the need for tight regulation (Fei et al., 2008; Heit et al., 2006; Bernal-Mizrachi et al., 2010).

In addition, lipotoxic conditions modify granule composition, leading to a shift from a normal biphasic Ca^2+^ response to a monophasic high-affinity Ca^2+^ profile, indicating disrupted Ca^2+^-dependent exocytosis (Kreutzberger et al., 2020).

Together, these findings emphasize Ca^2+^ dysregulation as a central mechanism of β-cell dysfunction in T2D.

### 7.1 Multi-organelle Ca^2+^ and T2D

Several Ca^2+^ signaling components from different organelles and intracellular compartments are altered in β-cells exposed to T2D-mimicking conditions and in islets from animal models and individuals with T2D.

Disruption in ER Ca^2+^ handling represent a central feature of β-cell dysfunction. Defects in ER Ca^2+^ release from RyR channels play an important role in diabetes pathophysiology, as their expression or function is reduced in diabetic animal models (Islam, 2002) and human islets (Patti et al., 2003). RyRs contribute to insulin secretion in human and mouse islets (Islam, 2002), and their inhibition prevents tunicamycin-induced dysfunctional Ca^2+^ oscillations and β-cell death (Yamamoto et al., 2019). Cytokines and ER stress inducers differentially affect ER Ca^2+^ channels, as cytokines impair IP_3_R function, whereas ER stress inducers preferentially promote RyR-mediated Ca^2+^ leak (Yamamoto et al., 2019; Zhang et al., 2023). In parallel, SERCA2b expression is reduced in db/db mice and human diabetic islets (Kono et al., 2012), leading to decreased ER Ca^2+^ content, impaired Ca^2+^ oscillations, and reduced GSIS (Liang et al., 2014). Blocking SERCA pumps in mouse MIN6 β-cells and mouse islets leads to depletion of ER Ca^2+^, ER stress and cell death (Luciani et al., 2009). Although inhibition of IP_3_R or RyR alone does not trigger death, their activity influences the severity of ER stress and apoptosis caused by SERCA dysfunction (Luciani et al., 2009). While SERCA3 KO mice remain normoglycemic and normoinsulinemic (Arredouani et al., 2002), rare SERCA3 mutations in humans associate with hyperglycemia and β-cell dysfunction in normal-weight individuals (Varadi et al., 1999).

Mitochondrial dysfunction is another key component of altered Ca^2+^ homeostasis in T2D. Altered mitochondrial morphology and respiration are altered in β-cells from human patients with T2D (Del Guerra et al., 2005; Anello et al., 2005), contributing to age-dependent decline in insulin secretion in human islets, a defect also observed in aged mouse islets (Gregg et al., 2016). In mice, compensatory mechanisms include reduced K_ATP_ channel conductance and increased β-cell sensitivity (Gregg et al., 2016). Notably, mice lacking Abcc8, a K_ATP_ subunit, exhibit chronic β-cell depolarization and elevated [Ca^2+^]_i_, which, when combined with a HFD, exacerbates mitochondrial dysfunction, and impairs β-cell function (Osipovich et al., 2020). In models of progressive insulin resistance, β-cells present mitochondrial swelling, reduced mitochondrial Ca^2+^ uptake, impaired Ca^2+^ signaling, and diminished GSIS (Lu et al., 2009).

Interestingly, impairment of mitochondrial function may also compromise the function of other organelles, such as the ER. For instance, pharmacological impairment of mitochondrial function has been shown to increase ER stress markers, in association with increased apoptosis in mouse MIN6 β-cells (Lee et al., 2010). Additionally, induction of mitochondrial fission also induces ER stress and apoptosis of rat INS-1 β-cells (Peng et al., 2011). Conversely, ER stress and Ca^2+^ dysregulation further impair mitochondrial function, highlighting a bidirectional relationship.

As already indicated, at the interface between these organelles, MAM microdomains mediate ER-mitochondria Ca^2+^ exchange, essential for proper GSIS. Acute glucose stimulation enhances ER-mitochondria interactions in rat INS-1E β-cells, while its disruption impairs GSIS (Dingreville et al., 2019). In contrast, chronic exposure to high glucose increases organelle interactions, but alters this coupling, reducing ER Ca^2+^ stores, increasing basal mitochondrial Ca^2+^ levels, and impairing ATP-stimulated ER-mitochondria Ca^2+^ exchange (Dingreville et al., 2019). Pharmacological SERCA activation increases ER and mitochondrial Ca^2+^ levels, improving mitochondrial bioenergetics and insulin secretion (Nguyen et al., 2023). Moreover, under PA exposure, SERCA activation preserves ER-mitochondria function and prevents mitochondrial dysfunction, oxidative stress, and β-cell death (Nguyen et al., 2023).

Consistent with this, β-cells from individuals with T2D and mouse MIN6 β-cells exposed to PA exhibit reduced ER-mitochondria interactions and altered GSIS (Thivolet et al., 2017). Furthermore, upregulation of PDZD8, a key MAM-associated protein, is observed in islets from HFD-fed mice and in rat INS-1 β-cells treated with PA (Liu et al., 2024). Silencing PDZD8 in rat INS-1 β-cells leads to decreased expression and interaction of MAM proteins, reduced expression of ER stress markers and ER stress, and decreases Ca^2+^ flow into the mitochondria, culminating in decreased cell death (Liu et al., 2024). Recent studies also identify MAM-related biomarkers associated with β-cell dysfunction in T2D, supporting the relevance of ER-mitochondria interactions in T2D pathophysiology and pointing out to a potential as therapeutic targets (Li et al., 2025).

Metabolic stress further integrates these alterations. Exposure of human islets to glucolipotoxic conditions induces great transcriptome modifications, with upregulation of several processes, including the Unfolded Protein Response (UPR), protein degradation pathways, autophagy and ER stress-induced apoptosis, and persistent β-cell dysfunction even after stress removal (Marselli et al., 2020). Disturbances in ER Ca^2+^ homeostasis can potentially lead to UPR activation, which is observed in islets from T2D patients and animal models (Zhang et al., 2020; Back and Kaufman, 2012). Pro-inflammatory cytokines, such as IL-1β, induce β-cell death through ER Ca^2+^ depletion, ER stress and mitochondrial dysfunction (Wang et al., 2009; Verma et al., 2013).

Together, these findings demonstrate that dysregulation of Ca^2+^ signaling across the ER, mitochondria, and ER-mitochondria interactions is a central mechanism in β-cell dysfunction in T2D.

## 8 ROS: oxidative stress and antioxidant defenses in T2D

As already discussed, ROS play an important role in GSIS as signaling molecules. However, the effects of ROS are highly context-dependent. While physiological and transient ROS generation is required for GSIS, sustained or excessive ROS levels promote oxidative stress and β-cell failure, contributing to T2D development.

Both glucotoxicity and lipotoxicity are major sources of ROS in β-cells. Several studies have investigated the effects of fatty acids, mainly saturated, on ROS production in isolated islets and β-cell lines leading to oxidative stress and β-cell death. Chronic exposure of rat INS-1 832/13 β-cells to high glucose and/or PA leads to impaired GSIS, increased ROS levels and altered expression of antioxidant enzymes (Fu et al., 2017). Moreover, exposure of β-cells to high glucose and PA impairs mitochondrial respiration, increases mitochondrial ROS, reduces GSIS, and decreases cell viability (Barlow and Affourtit, 2013). PA also induces H_2_O_2_ formation, ER stress and β-cell toxicity (Gehrmann et al., 2015).

In contrast, unsaturated FFAs can exert partially protective effects. Palmitoleate prevents PA-induced ROS accumulation and cell death, without restoring mitochondrial respiration or insulin secretion (Barlow and Affourtit, 2013), and oleate prevents PA-induced H_2_O_2_ production (Gehrmann et al., 2015). However, this protection may be context-dependent, as chronic exposure of human islets to oleate alone reduces GSIS and insulin content, and increases ROS production, indicating oxidative stress (Bikopoulos et al., 2008). Supporting these findings, intravenous oleate infusion in mice increases ROS production, lipid peroxidation, and impairs β-cell function, whereas GPx4-overexpressing protects from all these detrimental effects of oleate (Koulajian et al., 2013).

Of note, emerging evidence suggests that ROS also contribute to early β-cell dysfunction before overt impairment in GSIS (Corkey et al., 2021). In metabolic conditions such as obesity and T2D, where both lipid availability and oxidative state are elevated, failure to properly resolve ROS signaling may lead to its sustained elevation. Sustained ROS elevation in the presence of excess lipid availability may drive basal insulin hypersecretion prior to GSIS impairment through interactions with lipid-derived signals such as long-chain acyl-CoA esters (LC-CoA) (Corkey et al., 2021). This process can occur in the absence of glucose, and thus without a proportional increase in insulin biosynthesis. This imbalance can progressively deplete insulin stores and contribute to β-cell exhaustion. In this framework, basal hyperinsulinemia may initially represent an adaptive response to nutrient excess, but becomes maladaptive when chronic demand exceeds β-cell capacity, thereby linking early redox and lipid signaling dysregulation to later β-cell failure.

Importantly, oxidative stress is determined by a delicate balance between ROS production and the efficiency of antioxidant defense systems. These processes are spatially organized, as both ROS production and antioxidant defenses vary across subcellular compartments, shaping local redox balance and susceptibility to oxidative stress (Roma and Jonas, 2020). This balance also defines the threshold between physiological redox signaling and pathological oxidative stress in β-cells. Factors such as the duration and magnitude of ROS exposure, the source of ROS production, the availability of NADPH, and the capacity of antioxidant systems to respond to metabolic and inflammatory stress all influence this transition. Acute glucose exposure reduces intracellular H_2_O_2_ levels and increases NAD(P)H (Deglasse et al., 2019), supporting redox buffering capacity. However, this contrasts with the deleterious effects observed under long-term exposure to high glucose, which impairs antioxidant capacity by reducing G6PD, an important source of NADPH (Zhang et al., 2010). Importantly, although β-cells possess efficient antioxidant systems, their ability to adapt to prolonged metabolic or inflammatory stress appears to be limited, particularly due to limitations in NADPH production and antioxidant upregulation. Consistently, G6PD overexpression restores β-cell function under glucotoxic conditions (Zhang et al., 2010).

NADPH plays a central role in maintaining redox homeostasis in β-cells. It supports antioxidant systems such as thioredoxin and glutathione, and also serves as a substrate for NOX, thereby contributing to ROS generation. Although classical sources such as the pentose phosphate pathway (PPP) contribute to NADPH production, in β-cells it is also produced through anaplerotic metabolism of glucose and other nutrients (Grubelnik et al., 2024). This dual role places NADPH at the intersection of metabolic flux, ROS production, and antioxidant defense, highlighting its importance in the regulation of redox balance under both physiological and diabetic conditions.

Various antioxidant systems modulate β-cell resilience. Stable expression of ER-resident GPx7 or GPx8 in rat INS-1E β-cells, which normally do not express these enzymes, attenuates PA-mediated H_2_O_2_ generation, ER stress, and apoptosis, and enhances GSIS (Mehmeti et al., 2017). Similarly, the antioxidant response mediated by NRF2 modulates β-cell sensitivity to ROS, as Nrf2 knockdown in mouse MIN6 β-cells or islets from Nrf2 KO mice have increased ROS accumulation and susceptibility to oxidative damage, whereas NRF2 activation protects β-cells from H_2_O_2_-induced cell death (Fu et al., 2015). This highlights that, while ROS are essential for GSIS, inadequate antioxidant defense exacerbates oxidative stress and β-cell dysfunction.

Despite their apparent vulnerability, β-cells can trigger adaptive antioxidant responses under certain conditions. In Goto-Kakizaki rats, islets develop increased glutathione levels and upregulate antioxidant genes, maintaining basal ROS levels comparable to or lower than controls and exhibiting resistance to oxidative stress (Lacraz et al., 2009). However, oxidative stress can also induce long-lasting dysfunction. A single transient exposure to H_2_O_2_ leads to persistent dysfunction of rat INS-1E β-cells, with reduced GSIS, impaired mitochondrial respiration, and increased mitochondrial ROS production lasting for several days (Li et al., 2009). Interestingly, previously stressed cells have partial resistance to a second oxidative insult, associated with UCP2 upregulation, suggesting the coexistence of sustained mitochondrial damage and adaptive protective responses (Li et al., 2009).

Intermittent metabolic stress also exacerbates oxidative damage. In rat INS-1 β-cells, intermittent high glucose exposure induces greater increases of [Ca^2+^]_i_ and ROS, apoptosis, and impaired insulin secretion compared to sustained high glucose, while pretreatment with an antioxidant significantly attenuates these responses (Shao et al., 2015). Similarly, in islets from Wistar rats intravenously infused with high glucose, co-infusion with some antioxidants partially protects from ROS production, ER stress activation and dysfunction (Tang et al., 2007; Tang et al., 2012). Early antioxidant intervention, such as N-acetylcysteine (NAC), delays hyperglycemia in Zucker Diabetic Fatty rats, and improves insulin secretion in T2D patients, although these protective effects may decline over time (Robertson et al., 2007).

Importantly, β-cells are more resilient to oxidative stress than initially supposed, as they can detoxify micromolar levels of H_2_O_2_ through the peroxiredoxin/thioredoxin system when exposure is continuous, which may better reflect physiological conditions than a single dose (Stancill et al., 2019). Of note, this defense has limits, since acute bolus of H_2_O_2_ induces DNA damage and decreases β-cell viability, and prolonged metabolic stress leads to cumulative dysfunction due to the limited adaptive capacity of antioxidant systems (Stancill et al., 2019).

Thus, β-cell redox biology is best understood as a dynamic balance between efficient handling of physiological ROS signals and progressive vulnerability under chronic stress conditions. In this context, ROS act as both signaling molecules and mediators of damage, and the balance between ROS production and antioxidant defenses is a key determinant of β-cell function in T2D (Holendov et al., 2024).

### 8.1 ROS sources in T2D

As discussed above, ROS are produced by multiple intracellular sources. In the context of obesity and T2D, oxidative stress originates from the combined contribution of several organelles and compartments, notably mitochondria, NOXs, peroxisomes and the ER.

Mitochondria represent a major source of ROS in β-cells in the context of T2D. PA-induced ROS production has been shown to promote β-cell dysfunction and death (Barlow and Affourtit, 2013). Further analyses suggest that this ROS production is primarily mitochondrial, as MitoSOX oxidation increases while cytosolic ROS levels remain unchanged (Barlow et al., 2015). In line with this, exposure of rat INS-1 832/13 β-cells to high glucose and PA impairs mitochondrial function, increases ROS production, and reduces GSIS (El-Assaad et al., 2010). Mechanistically, mitochondrial ROS may impair β-cell function through activation of UCP2, as high glucose increases UCP2 expression and mitochondrial superoxide levels, leading to impaired GSIS, similar to islets from ob/ob mice (Krauss et al., 2003). Notably, UCP2 KO preserves GSIS (Krauss et al., 2003).

Various studies also support a role for NOX enzymes in β-cell dysfunction. PA reduces GSIS in mouse islets, an effect prevented by antioxidant treatment or NOX inhibition with apocynin (Michalska et al., 2010). Further evidence in β-cell lines also shows that chronic hyperglycemia induces NOX activity, which is attenuated by apocynin (Mohammed and Kowluru, 2013).

Specific NOX isoforms may also play a role, depending on the context. We have recently shown that NOX isoforms are distributed in β-cells across the ER, insulin vesicles, and plasma membrane, providing multiple sources of ROS (De Almeida et al., 2025). We also show that inflammatory cytokines alter the expression of specific NOX isoforms in a time-dependent manner, suggesting isoform-specific roles in ER and mitochondrial stress, ROS production, and β-cell death (De Almeida et al., 2025).

NOX1 expression is significantly increased in islets from individuals with T2D (Weaver et al., 2012), and its activation by cytokines impairs GSIS, effects that are prevented by NOX1 inhibitors (Weaver et al., 2015; Weaver and Taylor-Fishwick, 2017). Moreover, NOX1 overexpression recapitulates cytokine-induced ROS production, loss of GSIS, and apoptosis, while NOX1 knockdown protects against these deleterious effects (Weaver and Taylor-Fishwick, 2017). NOX2 inhibition significantly reduces high glucose-induced NOX2 activation and ROS production (Sidarala et al., 2015), while NOX2 silencing protects from ROS production and GSIS decrease in β-cells exposed to high glucose (Yuan et al., 2010b) and PA and oleate (Yuan et al., 2010a). Using genetically encoded H_2_O_2_ sensors, our group has demonstrated that PA induces an early and transient NOX2-dependent ROS production, leading to β-cell dysfunction and death (Vilas-Boas et al., 2021b). Consistently, PA exposure increases NOX activity and expression of NOX2 and its subunit p47_phox_, with p47_phox_ silencing protecting against ROS production and GSIS impairment (Sato et al., 2014). Lipoproteins, such as LDL and VLDL, similarly induce NOX2-dependent ROS production, leading to reduced insulin secretion and increased apoptosis (Jiao et al., 2012). NOX4 has also emerged as a potential therapeutic target, as its inhibition improves glucose tolerance and protects against β-cell death in both mouse and human islets (Anvari et al., 2015).

The regulation of NOX activity is also relevant, as upstream signaling molecules such as the small GTPase Rac1 controls NOX-induced ROS production in β-cells. High glucose activates Rac1 in β-cells, which stimulates NOX activity, increases ROS production, induces mitochondrial dysfunction, and promotes apoptosis (Elumalai et al., 2017). Similarly, PA induces Rac1 activation, ROS production, and lipid peroxidation, effects attenuated by inhibition of NOX or Rac1 (Syed et al., 2010). In diabetic models and human islets, Rac1 activation correlates with increased ROS production and apoptotic signaling (Syed et al., 2011).

In addition to mitochondrial and NOX-derived ROS, other organelles also contribute to ROS generation. Long-chain saturated FFAs induce H_2_O_2_ formation in peroxisomes (Gehrmann et al., 2015; Elsner et al., 2010). Overexpression of catalase in the cytosol or peroxisomes, but not in mitochondria, significantly reduces H_2_O_2_ levels and protects β-cells from PA-induced death (Elsner et al., 2010). In addition, PA induces H_2_O_2_ accumulation in the ER, contributing to ER stress and β-cell dysfunction (Mehmeti et al., 2017).

ROS production in different organelles is very interconnected. Studies using compartment-specific ROS sensors show that very long-chain FFAs strongly elevate H_2_O_2_ in peroxisomes and mitochondria, accompanied by ER stress and oxidative damage (Plötz et al., 2019). Moreover, oxidative stress can directly trigger ER stress, as oxidized LDL induces ER stress and β-cell death in a ROS-dependent manner (Plaisance et al., 2016). A crosstalk between mitochondria and the ER is critical for redox balance, as mitochondrial dysfunction alters cellular redox donor availability, leading to ER hyperoxidation and impaired proinsulin processing (Rohli et al., 2024). Interestingly, we have shown that attenuation of ER stress prevents subsequent NOX2 activation in mouse islets under inflammatory cytokine exposure, suggesting that ER stress may precede NOX2 activation (Vilas-Boas et al., 2021c).

Finally, altered ER redox homeostasis appears to be a critical mechanism in β-cell dysfunction, as hyperoxidation of the ER delays proinsulin export and limits insulin granule formation (Rohli et al., 2022).

Although multiple intracellular sources contribute to ROS production in β-cells under physiological contexts and metabolic stress, accumulating evidence suggests that these sources do not contribute equally or independently. Mitochondrial ROS appears to represent a primary driver in response to nutrient overload, closely linked to increased metabolic flux and electron transport chain activity. In parallel, NOX-derived ROS may act predominantly as required signaling molecules in GSIS (NOX4) and also as key ROS sources particularly under inflammatory and lipotoxic conditions (NOX1-2). Other compartments, such as the ER and peroxisomes, often function as modulators that both respond to and propagate oxidative stress.

Importantly, these sources form a highly interconnected redox network rather than isolated contributors. For instance, emerging evidence supports the existence of feed-forward loops in which initial stress signals, such as ER stress, may trigger NOX2 activation, further amplifying ROS production and exacerbating organelle dysfunction. However, the precise temporal hierarchy among these sources remains incompletely defined and may vary across different stages of T2D progression. A comprehensive overview of how alterations in redox homeostasis are associated with changes in GSIS under pathological β-cell conditions is summarized in Table 4.

**TABLE 4:** Alterations in ROS homeostasis associated with impaired glucose-stimulated insulin secretion (GSIS) under diabetogenic conditions. Summary of studies investigating how diabetogenic conditions, including glucotoxicity, lipotoxicity, obesity, and inflammation, disrupt ROS homeostasis and contribute to impaired GSIS in pancreatic β-cells.

Model	Condition	Effect	References
Mouse NIT-1 β-cells.	Free fatty acid-induced lipotoxicity.	Increased NOX2-derived ROS, impaired GSIS, and enhanced β-cell apoptosis.	Yuan et al. (2010a)
Rat pancreatic islets.	48-h glucose infusion (20 mmol/L) ± taurine (TAU), N-acetylcysteine (NAC), or tempol (TPO).	Prolonged hyperglycemia increased total and mitochondrial superoxide levels and impaired GSIS; tempol prevented superoxide accumulation and restored GSIS, whereas TAU and NAC failed to preserve β-cell function.	Tang et al. (2007)
Rat pancreatic islets.	48-h glucose infusion (20 mmol/L) ± ER stress-relieving agents (PBA or TUDCA) or tempol (TPO).	ER stress and mitochondrial superoxide accumulation impaired GSIS, whereas PBA, TUDCA, and TPO restored glucose-induced insulin secretion.	Tang et al. (2012)
Rat INS-1 β-cells.	Prolonged glucotoxicity and lipotoxicity.	Reduced GSIS associated with chronic oxidative stress, adaptive antioxidant response, impaired glucose-derived ROS signaling, and mitochondrial uncoupling.	Fu et al. (2017)
Rat INS-1E β-cells.	Chronic glucolipotoxicity.	Mitochondrial dysfunction, increased ROS production, impaired GSIS, and reduced cell viability.	Barlow and Affourtit (2013)
Human pancreatic islets.	Chronic exposure to oleate.	Increased ROS production accompanied by reduced insulin content and impaired GSIS.	Bikopoulos et al. (2008)
Mouse pancreatic islets.	Oleate exposure and GPx4 overexpression.	Lipid peroxidation-mediated oxidative stress impaired GSIS and β-cell function; GPx4 overexpression prevented lipid peroxidation and preserved GSIS.	Koulajian et al. (2013)
Rat pancreatic islets.	Acute glucose stimulation.	GSIS was associated with decreased cytosolic and mitochondrial H_2_O_2_ levels rather than increased ROS production.	Deglasse et al. (2019)
Rat INS-1E β-cells.	GPx7 or GPx8 overexpression.	Enhanced GSIS, associated with improved ER antioxidant capacity and reduced oxidative stress.	Mehmeti et al. (2017)
• Rat INS-1E β-cells. • Rat pancreatic islets.	Transient oxidative stress (H_2_O_2_ exposure).	Reduced GSIS, associated with persistent mitochondrial dysfunction, increased mitochondrial ROS production, reduced ATP generation, and impaired mitochondrial biogenesis.	Li et al. (2009)
Rat INS-1 β-cells.	Intermittent high glucose compared with sustained high glucose.	Increased ROS production, enhanced apoptosis, and impaired GSIS.	Shao et al. (2015)
Rat INS-1 832/13 β-cells.	Glucolipotoxicity (high glucose and palmitate exposure).	Increased ROS production, mitochondrial dysfunction, and impaired GSIS.	El-Assaad et al. (2010)
Pancreatic islets isolated from obese diabetic ob/ob mice and ob/ob UCP2^−/−^ mice.	Mitochondrial superoxide-induced UCP2 activation.	Increased oxidative stress, mitochondrial uncoupling, and impaired GSIS.	Krauss et al. (2003)
• Mouse pancreatic islets. • Rat BRIN-BD11 β-cells.	Pro-inflammatory cytokines, palmitate, or H_2_O_2_ exposure.	Increased NOX/iNOS signaling, reduced insulin secretion, and β-cell dysfunction.	Michalska et al. (2010)
• Human and mouse pancreatic islets. • Murine β-cell lines (βTC-3 and NIT-1).	Cytokine-induced inflammatory stress.	NOX1-derived ROS abolished GSIS, whereas NOX1 inhibition preserved insulin secretion and reduced β-cell apoptosis.	Weaver et al. (2015)
• MIN6 β-cells. • Mouse pancreatic islets.	Cytokine-induced inflammatory stress and modulation of NOX1 expression.	Increased NOX1-derived ROS impaired GSIS and promoted β-cell apoptosis, whereas NOX1 knockdown conferred protection.	Weaver and Taylor-Fishwick (2017)
• Rat INS-1 832/13 β-cells. • Rat pancreatic islets.	Chronic high-glucose exposure.	Activation of the Rac1-Nox2-ROS pathway impaired GSIS and promoted metabolic dysfunction.	Sidarala et al. (2015)
Mouse pancreatic islets.	Chronic palmitate exposure.	NOX2-derived H_2_O_2_ mediated lipotoxicity, impairing insulin secretion and Ca^2+^ homeostasis; NOX2 deletion prevented these effects.	Vilas-Boas et al. (2021b)
Rat INS-1D β-cells.	Palmitate-induced lipotoxicity.	Src-dependent activation of NADPH oxidase increased ROS production and impaired GSIS.	Sato et al. (2014)
• Mouse NIT-1 β-cells. • ApoCIII transgenic mice.	VLDL-induced lipotoxicity.	Increased NOX2-derived ROS impaired insulin secretion and induced β-cell apoptosis.	Jiao et al. (2012)
• Human pancreatic islets. • Mouse pancreatic islets.	Metabolic stress induced by palmitate and HFD.	NOX4 inhibition improved GSIS and attenuated ROS-mediated β-cell dysfunction.	Anvari et al. (2015)
Mouse pancreatic islets from WT and NOX2 KO mice.	Pro-inflammatory cytokine exposure.	ER stress-dependent NOX2-derived ROS impaired insulin secretion and promoted β-cell death; these effects were prevented by NOX2 deletion.	Vilas-Boas et al. (2021c)

## 9 Crosstalk of Ca^2+^ and ROS in β-cells

In the next sessions, we will discuss the main findings on the crosstalk of Ca^2+^ and ROS in GSIS and in T2D in pancreatic β-cells. This information is also summarized in Table 5, which provides a representative overview of studies linking alterations in Ca^2+^ and/or ROS homeostasis to changes in insulin secretion in pancreatic β-cells and islets. For clarity, the studies are categorized according to the experimental model used, followed by the experimental condition and the observed effect on GSIS under physiological, adaptive, and diabetic conditions.

**TABLE 5:** Crosstalk of Ca^2+^ and ROS in pancreatic β-cells. The table summarizes selected studies from recent years illustrating key mechanisms of interaction between Ca^2+^ and reactive oxygen species (ROS) in pancreatic β-cells and islets, under both physiological and pathological conditions.

​	​	Model	Crosstalk observation	References
Ca^2+^→ROS	Physiological/Pathological	• Rat pancreatic islets. • Rat INS-1E and BRIN-BD11 β-cell lines.	Ca^2+^-sensitive DUOX enzymes link intracellular Ca^2+^ signaling to ROS generation.	De Almeida et al. (2025)
	Pathological	Pancreatic islets from transgenic mice expressing human NOX5 in β-cells under high-fat diet.	Ca^2+^-sensitive NOX5-derived ROS contribute to dysfunction and impaired insulin secretion.	Bouzakri et al. (2020)
	Pathological	• Rat INS-1 β-cell line with targeted deletion of SEL1L (involved in ERAD pathway). • β-cell-specific SEL1L KO mice.	ERAD dysfunction impairs mitochondrial Ca^2+^ influx, disrupting intracellular Ca^2+^ signaling and mitochondrial redox homeostasis during GSIS.	Hu et al. (2019)
ROS→ Ca^2+^	Physiological	• Mouse Pancreatic islets. • Rat INS-1E β-cells exposed to glucose.	NOX4-derived H_2_O_2_ regulates K_ATP_ channel activity and sustains Ca^2+^ oscillations during GSIS.	Plecitá-Hlavatá et al. (2020)
	Physiological	• Isolated pancreatic islets. • Dissociated β-cells from rats.	H_2_O_2_ increases cytosolic Ca^2+^ *via* redox modification of RyR2 (S-glutathionylation), enhancing Ca^2+^ release and amplifying GSIS.	Llanos et al. (2015)
	Physiological	Isolated rat pancreatic islets.	mtROS generation promotes extracellular Ca^2+^ influx and GSIS; antioxidant treatment suppresses both Ca^2+^ mobilization and insulin secretion.	Leloup et al. (2009)
	Pathological	Rat pancreatic β-cells exposed to H_2_O_2_.	H_2_O_2_-induced oxidative stress alters intracellular Ca^2+^ dynamics and impairs β-cell function.	Nakazaki et al. (2000)
	Pathological	• Rat INS-1 832/13 β-cell line. • Mouse pancreatic islets. Exposed to ROS-related stressors (palmitic acid, chronic hyperglycemia, H_2_O_2_).	Reduced SERCA2b expression, causing ER Ca^2+^ depletion, ER stress, and β-cell death.	Hara et al. (2014)
	Pathological	Rat INS-1 832/13 β-cell line.	Palmitic acid induces ER Ca^2+^ depletion and increases cytosolic and mitochondrial Ca^2+^ ER-targeted H_2_O_2_ detoxification (*via* GPx8 or catalase) preserves Ca^2+^ homeostasis.	Sharifi et al. (2023)
	Pathological	β-cell-specific KO mice of mitochondrial oxidative phosphorylation complexes I, III, or IV.	Mitochondrial ROS overproduction Impaired GSIS Altered mitochondrial Ca^2+^ responses to glucose Upregulation of antioxidant enzymes.	Lang et al. (2023)
	Pathological	• Rat INS-1 and INS-1 832/13 cells β-cell lines. • TRPM2-KO Mouse pancreatic islets (WT and). • Human pancreatic islets.	ROS activates Ca^2+^-permeable TRPM2 channels, leading to dysregulation of intracellular Ca^2+^ homeostasis.	Reviewed in: (Malko et al., 2021)
	Pathological	Rat RINm5F β-cell line.	Tert-butyl hydroperoxide-induced oxidative stress causes membrane depolarization and leads to Ca^2+^ influx.	Wahl et al. (2009)
	Pathological	Rat INS-1E pancreatic β-cells exposed to hyperglycaemia.	Mitochondrial ROS disrupt Ca^2+^ homeostasis and promote ER stress, which is attenuated by MitoQ treatment.	Escribano-Lopez et al. (2019)

### 9.1 Ca^2+^ and ROS in GSIS

As discussed in previous sections, Ca^2+^ and ROS are two key signaling molecules in insulin secretion, acting in a tightly interconnected way. In β-cells, ROS can affect Ca^2+^ dynamics, and Ca^2+^ dynamics can influence oxidant production, establishing a bidirectional signaling network that fine-tunes insulin secretion.

Reinforcing this dynamic interplay in β-cells, studies in islets from Wistar rats have shown that H_2_O_2_ increases intracellular Ca^2+^ both from ER stores and *via* plasma membrane influx, in addition to promoting the closure of K_ATP_ channels (Nakazaki et al., 2000). Similarly, in isolated rat islets, exogenous H_2_O_2_ elevates cytosolic Ca^2+^, and the inhibition of RyR channels or treatment with the antioxidant NAC suppresses GSIS and prevents the H_2_O_2_-induced Ca^2+^ rise, emphasizing the important interplay between ROS and Ca^2+^ signaling in insulin secretion (Llanos et al., 2015). Other studies have demonstrated that ROS can modulate Ca^2+^ release from intracellular stores by regulating receptors such as IP_3_R and RyR, thereby contributing to increased cytosolic Ca^2+^ levels (Bánsághi et al., 2014; Llanos et al., 2015). Additional redox-sensitive channels, such as TRPM2, may further contribute to ROS-dependent Ca^2+^ entry (Görlach et al., 2015; Hara et al., 2002). These findings point to ROS as upstream modulators of Ca^2+^ dynamics, promoting ER Ca^2+^ release and plasma membrane influx through redox-sensitive targets.

At the subcellular level, at the MAM microdomain, Ca^2+^ and ROS signals are integrated. In these regions, IP_3_R-mediated Ca^2+^ release generates localized Ca^2+^ hotspots that promote mitochondrial Ca^2+^ uptake *via* MCU, enhancing metabolic activity and ROS production. This spatial organization enables precise coupling between Ca^2+^ fluxes and redox signaling (Görlach et al., 2015; Rizzuto et al., 2009; Hempel and Trebak, 2017). Such compartmentalization is essential for signaling specificity, allowing localized ROS to selectively modulate nearby Ca^2+^ channels and transporters (Murphy, 2009; Görlach et al., 2015).

Conversely, Ca^2+^ also regulates ROS production. Some NOX isoforms, such as NOX5, which has been detected in human pancreatic islets (Bánfi et al., 2001; Bouzakri et al., 2020), and DUOXs (De Almeida et al., 2025) are Ca^2+^-sensitive. In mitochondria, Ca^2+^ uptake by MCU stimulates TCA cycle dehydrogenases (Denton, 2009), increasing NADH and FADH_2_ supply to the electron transport chain and enhancing electron leakage and ROS formation.

In general, Ca^2+^ influx is the primary trigger for insulin exocytosis, although full secretory capacity requires ROS signaling. Notably, ROS do not act merely as byproducts of metabolism but, as already mentioned, as signaling molecules with a dual and context-dependent role in β-cell physiology. While low to moderate levels of ROS are required for proper stimulus-secretion coupling (Pi et al., 2007), excessive ROS impairs GSIS and disrupts Ca^2+^ dynamics.

**FIGURE 1:**
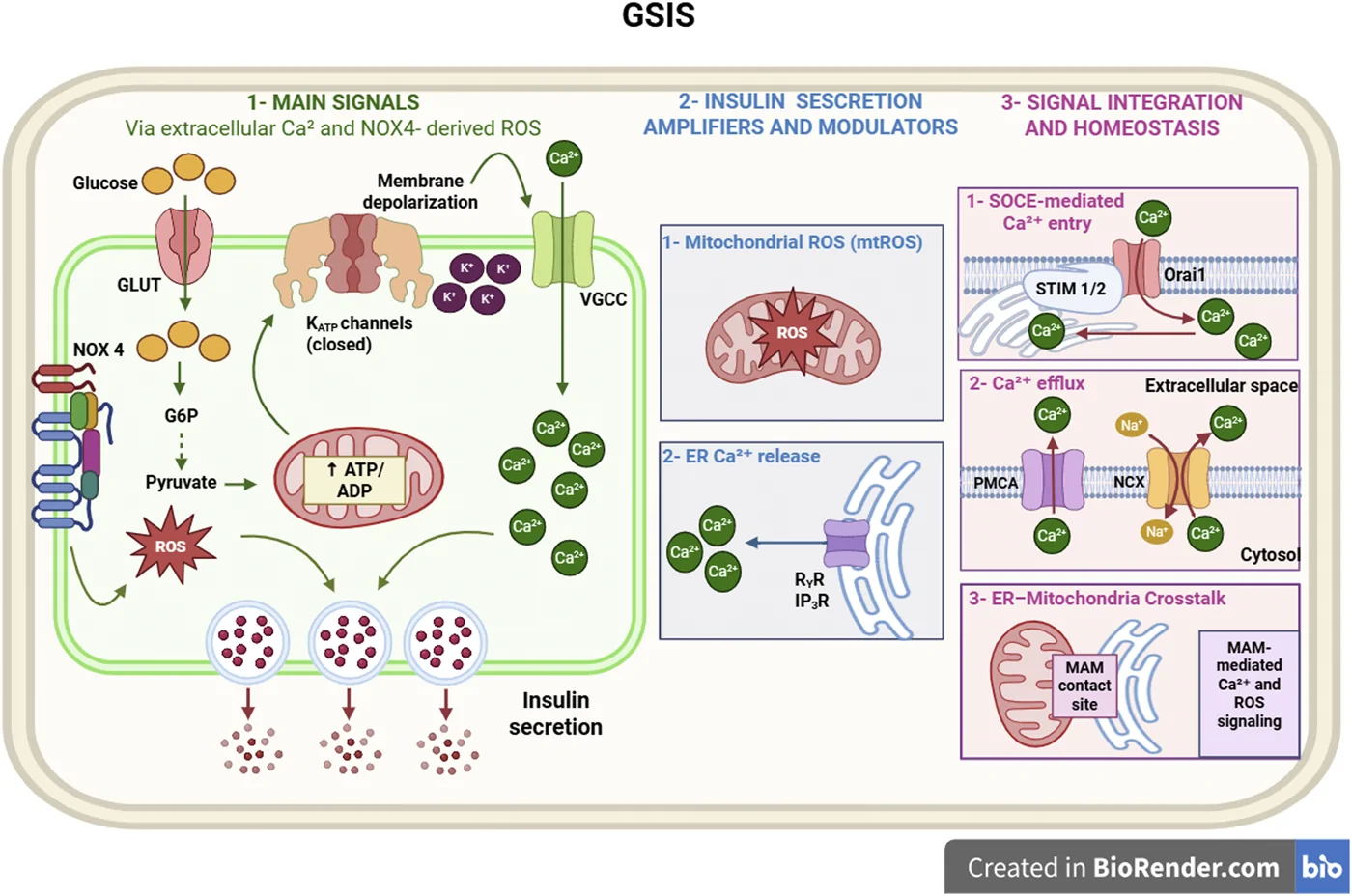
Sources of calcium (Ca^2+^) and reactive oxygen species (ROS) and their influence on glucose-stimulated insulin secretion (GSIS) in pancreatic β-cells. The figure is organized into three modules (1). Main signals: Glucose uptake *via* GLUT is followed by glycolysis and mitochondrial metabolism, increasing the ATP/ADP ratio. This promotes closure of ATP-sensitive K^+^ (K_ATP_) channels, membrane depolarization, and opening of voltage-gated Ca^2+^ channels (VGCCs), leading to Ca^2+^ influx. In parallel, NADPH oxidase (NOX4)-derived ROS is also required for proper insulin secretion (2). Insulin secretion amplifiers and modulators: Mitochondrial ROS (mtROS) and Ca^2+^ release from the endoplasmic reticulum (ER), mediated by inositol 1,4,5-trisphosphate receptors (IP_3_R) and ryanodine receptors (RyR), amplify cytosolic Ca^2+^ signals and insulin granule exocytosis (3). Signal integration and homeostasis: Ca^2+^ homeostasis is maintained by multiple mechanisms, including store-operated Ca^2+^ entry (SOCE), in which ER Ca^2+^ depletion is sensed by STIM1/2, activating Orai1 channels and promoting Ca^2+^ influx and ER Ca^2+^ replenishment; Ca^2+^ extrusion *via* plasma membrane Ca^2+^-ATPase (PMCA) and Na^+^/Ca^2+^ exchanger (NCX); and functional crosstalk between ER and mitochondria at mitochondria-associated membranes (MAMs), which coordinate Ca^2+^ transfer and ROS signaling. Together, these pathways integrate metabolic and redox signals to regulate GSIS. Created with BioRender.com.

In sum, under physiological conditions, the Ca^2+^/ROS signaling cascade follows a coordinated and dynamic sequence of events (Figure 1). Glucose uptake and metabolism increase the ATP/ADP ratio, leading to K_ATP_ channel closure, plasma membrane depolarization, and opening of VGCCs, thereby promoting Ca^2+^ influx and triggering insulin granule exocytosis. Notably, NOX4-derived H_2_O_2_ acts as a critical signal within this cascade, ensuring the efficiency of Ca^2+^-dependent insulin secretion through reversible oxidation of cysteine residues in target proteins, potentially by modulating key components of the stimulus-secretion coupling machinery, including K_ATP_ and Ca^2+^ channels. These sequential events are the main mechanisms underlying insulin secretion.

The increase in cytosolic Ca^2+^ is amplified by Ca^2+^ release from the ER *via* IP_3_R and RyR, which may also be regulated by redox- and metabolically sensitive mechanisms. In parallel, Ca^2+^ transfer from the ER to mitochondria at MAMs stimulates oxidative metabolism and controlled mitochondrial ROS production, which contributes to metabolic amplification of insulin secretion. Of note, Ca^2+^ may also contribute to ROS generation through Ca^2+^-sensitive NOX isoforms, although this mechanism is not considered a primary driver in β-cell stimulus-secretion coupling.

Finally, Ca^2+^ homeostasis is restored through coordinated extrusion and reuptake mechanisms, including SOCE to replenish ER Ca^2+^ stores, and Ca^2+^ extrusion *via* plasma membrane transporters such as NCX and PMCA. Also, intracellular antioxidant systems spatially and temporally restrict ROS accumulation, ensuring that redox signals remain reversible and compatible with physiological signaling.

Disruption of this coordinated Ca^2+^/ROS signaling network emerges as a key determinant of β-cell dysfunction and impaired insulin secretion in T2D.

### 9.2 Ca^2+^ and ROS in T2D

In obesity and T2D, chronic nutrient overload and hyperglycemia disrupt mitochondrial and ER Ca^2+^ homeostasis and increase ROS production from multiple sources, including mitochondria, NOXs, peroxisomes, and the ER, collectively triggering ER stress, inflammatory signaling, and β-cell dysfunction that impairs GSIS. Thus, the interaction between different stress pathways, for instance oxidative stress and Ca^2+^ signaling dysfunction, may play a key role.

As already mentioned in previous sections, ROS have a dual role in β-cell function, being both concentration- and context-dependent. Previous studies in rat INS-1 832/13 β-cells have shown that ROS-associated stressors, such as PA, chronic hyperglycemia, and H_2_O_2_, promote ER Ca^2+^ depletion and downregulation of SERCA2b, contributing to ER stress and β-cell death (Hara et al., 2014). These findings support ER Ca^2+^ depletion as an early event linking oxidative stress to β-cell dysfunction.

Long-term exposure of rat INS-1E β-cells to high glucose impairs GSIS, increases mitochondrial ROS production and intracellular Ca^2+^, and upregulates ER stress and NFκB-p65 (Escribano-Lopez et al., 2019). Treatment with a mitochondria-targeted antioxidant (MitoQ) prevented these hyperglycemia-induced detrimental effects (Escribano-Lopez et al., 2019). These results point to mitochondrial ROS as a key mediator connecting metabolic stress to Ca^2+^ dysregulation and impaired GSIS. In the same cell line, PA was shown to deplete ER Ca^2+^ and increase cytosolic and mitochondrial Ca^2+^ levels, leading to mitochondrial dysfunction and reduced ATP production (Sharifi et al., 2023). Importantly, detoxification of ER luminal H_2_O_2_, either through expression of the ER-resident GPx8 or ER-targeted catalase, preserves ER, cytosolic and mitochondrial Ca^2+^ homeostasis and insulin content (Sharifi et al., 2023). This suggests that localized ROS accumulation within the ER critically impacts Ca^2+^ handling and β-cell function.

In line with this, ER-associated degradation (ERAD) deficiency (by pharmacological inhibition or KO) impairs GSIS and is associated with reduced cytosolic Ca^2+^, increased mitochondrial Ca^2+^ and mitochondrial ROS (Hu et al., 2019). Importantly, inhibition of mitochondrial Ca^2+^ uptake prevents mitochondrial ROS overproduction (Hu et al., 2019), supporting a link between mitochondrial Ca^2+^ overload, mitochondrial ROS and β-cell dysfunction. Moreover, β-cell-specific complex III KO in mice caused early hyperglycemia, impaired GSIS, and disrupted mitochondrial Ca^2+^ responses to glucose (Lang et al., 2023). This was accompanied by increased expression of mitochondrial antioxidant enzymes (Pdx3, SOD2 and catalase) (Lang et al., 2023), indicating that excessive mitochondrial ROS generation contributes to Ca^2+^ dysregulation and β-cell failure.

Emerging evidence suggests that ROS can activate Ca^2+^-permeable TRPM2 channels in β-cells, leading to dysregulation of intracellular Ca^2+^ homeostasis (Malko et al., 2021). This ROS-TRPM2-Ca^2+^ axis seems to contribute to mitochondrial fragmentation, dysfunction, and the activation of apoptotic pathways. Interestingly, in rat RINm5F β-cells, oxidative stress induced by tert-butyl hydroperoxide promotes membrane depolarization, leading to Ca^2+^ influx and increased insulin secretion (Wahl et al., 2009), suggesting that redox-sensitive Ca^2+^ channels represent an additional layer through which ROS can modulate β-cell excitability and insulin secretion.

**FIGURE 2:**
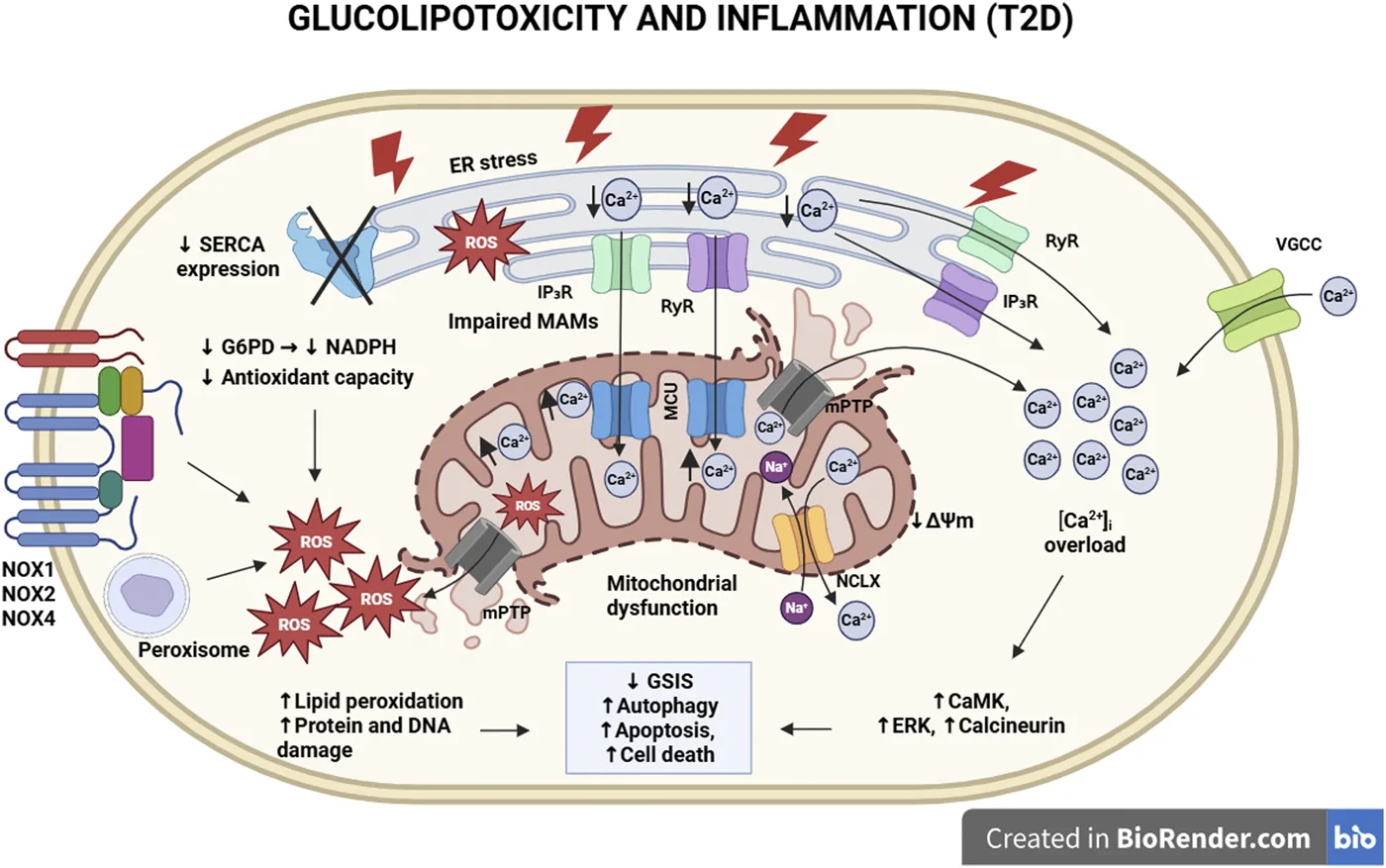
Disruption of Calcium (Ca^2+^) and Reactive Oxygen Species (ROS) homeostasis under glucolipotoxicity and inflammatory conditions in type 2 diabetes (T2D). Glucolipotoxicity and inflammation profoundly impair β-cell Ca^2+^ signaling and redox balance, contributing to dysfunction in obesity, prediabetes, and T2D. Under diabetic conditions, Ca^2+^ homeostasis is disrupted by increased Ca^2+^ influx through voltage-gated Ca^2+^ channels (VGCCs), together with impaired endoplasmic reticulum (ER) and mitochondrial Ca^2+^ handling. ER Ca^2+^ depletion occurs due to reduced sarco/endoplasmic reticulum Ca^2+^-ATPase (SERCA) activity, leading to loss of Ca^2+^ oscillatory control and cytosolic Ca^2+^ overload. ER Ca^2+^ release is further altered by reduced function of inositol 1,4,5-trisphosphate receptors (IP_3_R) and ryanodine receptors (RyR), contributing to ER stress. Mitochondrial Ca^2+^ uptake is also compromised, resulting in reduced bioenergetic efficiency and decreased mitochondrial membrane potential. At mitochondria-associated membranes (MAMs), disrupted ER-mitochondria communication impairs Ca^2+^ transfer and ATP production, while altered expression of MAM-associated proteins contributes to ER stress. Mitochondrial Ca^2+^ overload can induce opening of the mitochondrial permeability transition pore (mPTP), leading to loss of membrane potential and increased cytosolic release of Ca^2+^ and ROS. Downstream Ca^2+^-dependent signaling pathways, including Ca^2+^/calmodulin-dependent protein kinase (CaMK), extracellular signal-regulated kinases 1 and 2 (ERK1/2), and calcineurin, are dysregulated. In parallel, ROS homeostasis is similarly disrupted through increased production from mitochondria, NADPH oxidases (NOX1, NOX2, NOX4), peroxisomes, and the ER. Mitochondrial ROS generation is enhanced by electron transport chain dysfunction and reverse electron transport, contributing to oxidative stress and impaired ATP production. Reduced glucose-6-phosphate dehydrogenase (G6PD) activity decreases NADPH availability and weakens antioxidant defenses, further amplifying ROS accumulation. ROS promote lipid peroxidation and DNA damage. Together, ROS overproduction and Ca^2+^ dysregulation synergistically impair GSIS and promote autophagy, apoptosis, and β-cell death. Created in BioRender.com.

Thus, during T2D progression, these alterations evolve at multiple and interconnected levels, often beginning before overt hyperglycemia (Figure 2). Chronic nutrient overload, glucolipotoxicity, and inflammation perturb both redox balance and Ca^2+^ homeostasis, which seem to be intrinsically coupled from early stages of β-cell stress. In these conditions, there is a sustained ROS overproduction from multiple sources, including mitochondria, NOX, the ER, and peroxisomes, while Ca^2+^ dynamics becomes progressively destabilized.

**FIGURE 3:**
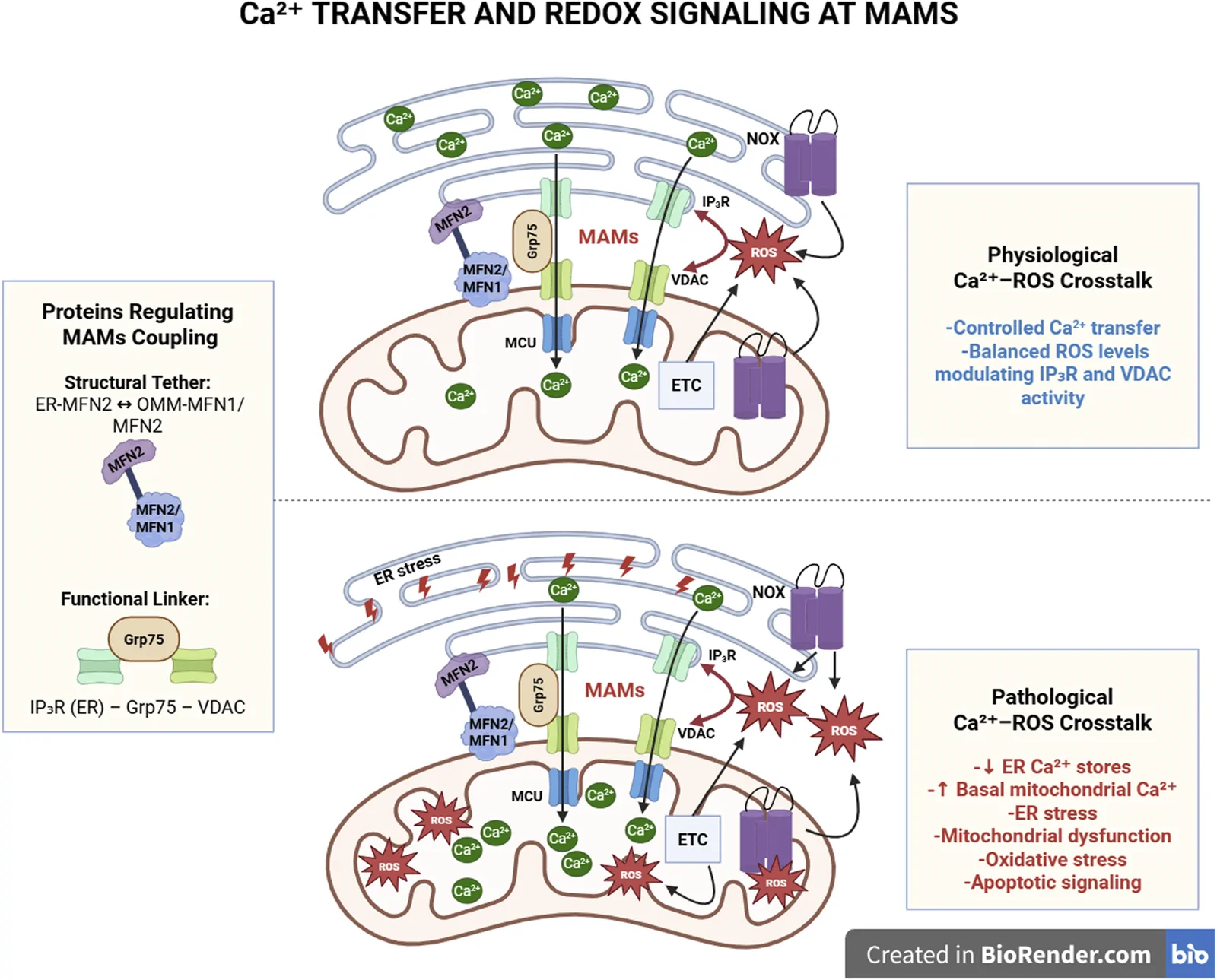
Mitochondria-associated membranes (MAMs) as platforms for Ca^2+^ transfer and Ca^2+^-ROS crosstalk. MAMs are specialized contact sites between the endoplasmic reticulum (ER) and mitochondria that facilitate interorganellar communication. Structural coupling is mediated by proteins involved in mitochondrial dynamics, particularly mitofusin 2 (MFN2), which localizes to the ER membrane and interacts with MFN1/MFN2 on the outer mitochondrial membrane to maintain ER–mitochondria contacts. Functional coupling is mediated by the IP_3_R–Grp75–VDAC complex, enabling Ca^2+^ transfer from the ER to mitochondria through the mitochondrial calcium uniporter (MCU). Under physiological conditions (top panel), tightly regulated Ca^2+^ transfer and controlled ROS production by NADPH oxidases (NOX) and the mitochondrial electron transport chain (ETC) modulate redox-sensitive targets, including IP_3_R and VDAC, thereby supporting Ca^2+^ signaling and cellular homeostasis. Under pathological conditions (bottom panel), increased ROS generation and dysregulated Ca^2+^ transfer promote ER stress, depletion of ER Ca^2+^ stores, and mitochondrial Ca^2+^ overload. This altered Ca^2+^-ROS crosstalk exacerbates oxidative stress, impairs mitochondrial function, and disrupts ER-mitochondria communication, ultimately contributing to β-cell dysfunction and apoptotic signaling. Red arrows indicate ROS-dependent modulation of IP_3_R and VDAC. Lightning symbols denote ER stress, while red starbursts represent sites of ROS accumulation.

While transient ROS is required for GSIS, prolonged or excessive ROS shifts from a signaling role to a deleterious one. A critical early transition occurs when ROS generation exceeds the capacity of antioxidant systems, leading to persistent oxidative imbalance. Particularly in the presence of excess lipid availability, elevated ROS can drive basal insulin hypersecretion independently of glucose, contributing to early β-cell stress and insulin depletion. As dysfunction progresses, excessive ROS disrupts Ca^2+^ homeostasis by impairing Ca^2+^ influx pathways (including VGCCs and redox-sensitive TRPM2) and by promoting ER Ca^2+^ depletion through altered IP_3_R and RyR activity and reduced SERCA expression, leading to ER stress. These alterations result in elevated basal cytosolic Ca^2+^ levels and loss of coordinated glucose-induced Ca^2+^ oscillations, which become poorly coupled to insulin exocytosis. Mitochondrial dysfunction, characterized by increased mitochondrial ROS, is exacerbated by Ca^2+^ overload due to impaired mitochondrial Ca^2+^ handling, reinforcing a feed-forward loop between mitochondrial Ca^2+^ accumulation and ROS generation that diminishes ATP production and uncouples metabolic signals from secretion. In parallel, disruption of ER-mitochondria communication at MAMs impairs Ca^2+^ transfer and metabolic coordination, while bidirectional crosstalk between ER stress and mitochondrial dysfunction amplifies oxidative stress and apoptotic signaling. These mechanisms converge to disrupt the tight coordination between metabolic, electrical, and secretory processes required for proper GSIS.

This progressive dysfunction is further aggravated by limited antioxidant adaptability, including impaired NADPH availability and insufficient upregulation of antioxidant systems, despite partial or transient compensatory responses. Thus, rather than isolated defects in individual pathways, the combined effects of Ca^2+^ dysregulation, oxidative stress, and mitochondrial and ER dysfunction culminate in defective insulin secretion and progressive β-cell failure.

Importantly, although these processes are highly interconnected, their precise causal hierarchy and temporal sequence during disease progression remain incompletely defined. In this context, emerging evidence suggests that specific stress pathways may exhibit a partial temporal hierarchy. For instance, our group has shown that, under inflammatory conditions, ER stress precedes NOX2 activation, identifying a potential early trigger for ROS amplification (Vilas-Boas et al., 2021c). However, whether such sequences are broadly conserved across different metabolic contexts remains unclear.

### 9.3 Intercellular coordination of Ca^2+^ dynamics and redox signaling in the islet microenvironment

Pancreatic islets are multicellular endocrine microorgans in which coordinated activity among distinct cell types is central for proper hormone secretion. β-cells display heterogeneous electrical activity, which needs to operate in a synchronized manner to generate coordinated intracellular Ca^2+^ oscillations and pulsatile insulin release (Gilon et al., 1993). This β-cell to β-cell synchrony relies on gap junctions composed of connexin 36 (Cx36) (Meda, 2012), allowing the exchange of ions and cytosolic molecules between neighboring β-cells. Disruption of Cx36-mediated coupling impairs intercellular Ca^2+^ synchronization, abolishes pulsatile insulin secretion, and leads to elevated basal insulin release, highlighting the importance of electrical and metabolic coupling for β-cell function (Ravier et al., 2005). Cx36 has been shown to be disrupted in diabetogenic states, with impaired network synchronization (Carvalho et al., 2012), although restoring coupling alone seems to be insufficient to fully recover coordinated islet function, indicating that additional regulatory mechanisms are involved (Levitt et al., 2026).

Beyond this, islet function is also shaped by specialized β-cell subpopulations. In this sense, early studies identified highly connected β-cell hubs as key coordinators of islet activity (Johnston et al., 2016), while more recent work has refined this view by revealing additional subsets, showing that hubs and wave-initiator cells are functionally distinct populations, with initiators triggering Ca^2+^ waves and hubs facilitating their propagation across the network (Stožer et al., 2022; Šterk et al., 2023). Of note, this coordinated network behavior is disrupted in T2D, with reduced Ca^2+^ wave propagation, impaired functional connectivity, and loss of global synchronization across β-cells (Gosak et al., 2022).

In addition to β-cell to β-cell coupling, other islet cell types also modulate this network dynamics: α-cells and δ-cells release glucagon and somatostatin, respectively, which regulate β-cell activity. In particular, glucagon binds to β-cell glucagon receptors (GCGR), enhances cAMP-dependent pathways and promotes Ca^2+^ mobilization and insulin secretion, linking paracrine inputs to β-cell excitability (Moede et al., 2020). Accordingly, β-cell loss of GCGR leads to reduced ability to propagate Ca^2+^ waves and to impaired insulin secretion. In contrast, somatostatin exerts inhibitory effects on α- and β-cells. Importantly, δ-cells do not merely act as passive inhibitors, but contribute to the fine-tuning of Ca^2+^ dynamics and hormone secretion within the islet network through combined paracrine signaling and gap junction-mediated interactions *via* Cx36 (Pourhosseinzadeh et al., 2025). Of note, this inhibitory tone is reinforced by β-cell-derived urocortin 3 (UCN3), which potentiates somatostatin secretion from δ-cells, thus indirectly suppressing α-cell Ca^2+^ and cAMP signaling and modulating glucagon release (Noguchi et al., 2024). It has been shown that Ca^2+^ coordination among different β-cells and between various cell types within the islet may be disrupted in models of diabetes, emphasizing the importance of intercellular communication (Gosak et al., 2022; Arrojo et al., 2019).

In parallel, the islet microenvironment is also shaped by redox signaling. ROS, particularly H_2_O_2_, may act as diffusible signaling molecules capable of crossing cellular membranes passively or facilitated by aquaporins with peroxiporin activity such as AQP8. In addition to direct diffusion, redox signal propagation may occur through redox relay mechanisms mediated by thiol-based proteins, enabling more specific and spatially controlled transmission of oxidative signals (Stöcker et al., 2018; Winterbourn, 2020).

Multiple sources can contribute to local signaling and the redox landscape of the islet. Importantly, plasma membrane-associated NOXs can generate extracellular ROS, enabling autocrine and paracrine signaling within the islet microenvironment and further extending their role beyond the β-cell itself. While autocrine redox signaling is well established in regulating stimulus-secretion coupling in β-cells, the role of ROS in paracrine signaling within the islet is less defined. Under pathological conditions, such as metabolic stress and T2D, dysregulation of these interconnected pathways may lead to impaired network function. Increased NOX activity and ROS production can intensify redox-dependent intercellular signaling, potentially amplifying islet dysfunction. Thus, depending on their subcellular location, NOX-derived ROS can operate not only as intracellular second messengers but also as local mediators of intercellular communication within the islet microenvironment.

Emerging evidence suggests that redox signaling may contribute to Ca^2+^ oscillation synchrony through the islet. Redox-dependent modifications of ion channels, enzymes, and gap junction proteins may influence β-cell excitability and coupling efficiency. In particular, gap junctions containing Cx36 appear to be sensitive to redox modifications through cysteine residues (Retamal et al., 2016). Through these mechanisms, local redox tone may shape the propagation and synchronization of Ca^2+^ oscillations across the islet.

In addition, β-cells may communicate through vesicle-mediated pathways. Emerging evidence indicates that β-cells communicate *via* exosomes, including microRNA cargo capable of modulating insulin secretion in recipient β-cells and potentially regulating antioxidant and redox-related gene expression in target cells. Additional evidence also suggests that β-cells communicate stress-related signals to neighboring or distant cells, potentially influencing oxidative stress responses and cellular function. Although distinct from direct ROS signaling, this pathway may represent a complementary route for transmitting redox-related information within and beyond the islet.

Altogether, intercellular coordination within the islet emerges from the integration of electrical coupling, paracrine signaling, and redox-dependent communication. These mechanisms do not act in isolation, but converge to regulate β-cell excitability, shape Ca^2+^ oscillation dynamics, and ensure proper hormone secretion at the islet level. Disruption of this integrated signaling network, as observed under metabolic stress and in T2D, can compromise Ca^2+^ synchronization and impair coordinated hormone secretion, contributing to islet dysfunction. Importantly, the relative contribution of these mechanisms may differ across different species. Notably, human β-cells exhibit different oscillatory behavior and reduced electrical coupling in comparison to rodent islets (Cabrera et al., 2006; Rorsman and Ashcroft, 2018), and this may affect how gap junctions and paracrine signaling contribute to islet coordination.

## 10 Concluding remarks

Taken together, the evidence discussed in this review supports the idea that Ca^2+^ and ROS are not independent regulators of β-cell function, but rather tightly interconnected signaling systems that coordinate insulin secretion under physiological conditions and become progressively dysregulated during the development of T2D. In the context of GSIS, Ca^2+^ influx through VGCCs represents the primary triggering signal for insulin secretion, while NOX-derived ROS, particularly NOX4-derived H_2_O_2_, act as essential redox signals that enable efficient stimulus-secretion coupling. In parallel, intracellular Ca^2+^ release from the ER functions as key amplifying mechanism that fine-tunes the efficiency and specificity of this process. In healthy β-cells, the coordinated Ca^2+^ and ROS oscillations integrate metabolic and electrical signals necessary for proper GSIS. On the other hand, chronic metabolic stress disrupts this coordination between multiple organelles, leading to sustained oxidative stress and disruption of Ca^2+^ signaling, defective interorganelle communication, and ultimately, β-cell failure.

Importantly, the available evidence suggests that not all components of the Ca^2+^/ROS network contribute equally throughout disease progression. Alterations in Ca^2+^ handling, mitochondrial dysfunction and ROS production from multiple sources (mitochondria, NOX, and the ER), together with disruption of antioxidant systems, contribute to the progressive disruption of cellular homeostasis in T2D. However, the precise temporal sequence and hierarchical relationships among these events remain incompletely understood.

Thus, despite substantial progress, several important mechanistic points remain poorly understood: (i) the temporal sequence linking ROS generation and Ca^2+^ dysregulation: whether oxidative stress primarily initiates Ca^2+^ defects, whether early alterations in Ca^2+^ handling drive ROS overproduction, or whether both are context-dependent; (ii) the relative contribution and hierarchy of various ROS sources (mitochondria, NOX isoforms, ER, and peroxisomes), especially at different stages of disease progression; (iii) how organelle-specific redox alterations shape local Ca^2+^ microdomains, as exemplified in MAMs; (iv) how β-cells define the threshold between physiological and pathological redox signaling and how this threshold is altered in T2D; (v) the role of NADPH-producing pathways in coordinating antioxidant defense and ROS generation; (vi) how early alterations causally contribute to late β-cell exhaustion and failure.

Answering these questions requires the development of robust experimental tools that allow for the assessment of Ca^2+^ and ROS dynamics in real time and with spatial resolution. In particular, the development of genetically encoded biosensors for Ca^2+^ and H_2_O_2_ targeting specific intracellular compartments (cytosol, ER, mitochondria, peroxisomes, MAM microdomains, *etc.*) is of great interest for dissecting organelle-specific signaling events in real time. Some of these sensors are already available and have contributed significantly to the beginning of our understanding of temporal responses. For example, using H_2_O_2_ sensors targeted to the nucleus/cytosol or mitochondrial matrix, we have characterized the temporal dynamics of H_2_O_2_ production in mouse islets under conditions mimicking T1D and T2D for up to 24 h. Combining these tools with live-cell imaging, targeted manipulation of ROS and Ca^2+^ pathways, and high-resolution mapping of interorganelle contacts will allow for a more integrated understanding of β-cell signaling networks. It is important to emphasize that transposing these approaches to human islets will be fundamental to validating the findings of experimental models.

Future studies should go beyond linear models of β-cell dysfunction and consider Ca^2+^ and ROS as components of a dynamic and multi-organellar signaling network. Incorporating β-cell heterogeneity and paracrine interactions within the islet will also be key for a comprehensive view. A systemic understanding of how metabolic, redox, and Ca^2+^ signals are integrated, and how this integration fails in T2D may aid in the development of pharmacological strategies to protect β-cell function.
